# Atypical Tetracyclines Promote Longevity and Ferroptotic Neuroprotection via Translation Attenuation

**DOI:** 10.1111/acel.70587

**Published:** 2026-06-18

**Authors:** Khalyd J. Clay, Manuel Sanchez‐Alavez, Ian Newman, Na Na, Ana P. Verduzco Espinoza, Alan To, Shannon Saad, Hollis T. Cline, Michael Petrascheck

**Affiliations:** ^1^ Department of Molecular and Cellular Biology The Scripps Research Institute La Jolla California USA; ^2^ Department of Neuroscience The Scripps Research Institute La Jolla California USA

**Keywords:** aging, ferroptosis, integrated stress response, longevity, neuroprotection, proteostasis, tetracyclines, translation inhibition

## Abstract

Reducing protein synthesis extends lifespan across taxa, but pharmacological strategies to safely attenuate translation remain limited. Tetracyclines are clinically used antibiotics long observed to exert beneficial effects in age‐associated diseases and extend lifespan in model organisms, though the underlying mechanisms remain unclear. Here, we systematically profiled commercially available tetracyclines and show that translation attenuation is a general property of the tetracycline class. Importantly, we identify the atypical tetracyclines 4‐epiminocycline and 12‐aminominocycline, which attenuate translation independently of antibiotic activity and integrated stress response (ISR) activation. These compounds extend lifespan in *C. elegans* , attenuate translation in human induced neurons, reduce hippocampal protein synthesis in vivo, and protect neurons from ferroptotic stress. Together, our results demonstrate that pharmacological attenuation of translation is sufficient to promote longevity and establish translation attenuation as a druggable longevity mechanism in mammals.

## Graphical abstract

Tetracyclines extend lifespan and protect neurons from ferroptosis by attenuating protein synthesis across multiple eukaryotic models through ISR‐dependent or ISR‐independent mechanisms, enabling classification based on their mode of action.

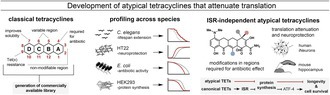

Conditions that reduce protein synthesis are among the most robust interventions known to extend lifespan across species (Hansen et al. 2007). Genetic inhibition of translation, inhibition of mTOR signaling, dietary restriction, and activation of stress response pathways all reduce protein synthesis and promote longevity (Anisimova et al. 2018). These interventions are thought to improve proteostasis by reducing the burden on protein folding and degradation systems, which decline with age (Hipp et al. 2019). However, pharmacological strategies to safely attenuate translation remain limited, particularly in old, post‐stress responsive animals where activation of stress responses such as the integrated stress response (ISR) may be impaired.

We previously identified minocycline, an FDA‐approved tetracycline antibiotic, to attenuate translation and extend lifespan, even in old, post‐stress responsive animals (Solis et al. 2018). The primary antibiotic mechanism of tetracyclines is the inhibition of the bacterial ribosome, preventing translation (Brodersen et al. 2000). Early studies comparing tetracycline activity in *E. coli* and *S. cerevisiae* showed that tetracyclines target mitochondrial ribosomes in yeast (Clark‐Walker and Linnane 1966). These findings, together with the ancestral origin of mitochondria (Chatzispyrou et al. 2015), provide a compelling narrative for their mechanism in eukaryotes. Indeed, doxycycline was shown to extend lifespan through a mechanism that involves the activation of the mitochondrial unfolded protein response (UPR^mt^) and the ISR (Mottis et al. 2022; Shao et al. 2025; Perry et al. 2021; Molenaars et al. 2020; Ronayne et al. 2023; Mortison et al. 2018; Brüning et al. 2014; Vendramin et al. 2021). However, unbiased chemoproteomics and biochemical methods have also shown that some tetracyclines bind the cytosolic ribosome to confer longevity through an ISR‐independent mechanism (Solis et al. 2018; Osterman et al. 2020; Protasoni et al. 2018). More broadly, genetic inhibition of translation is well established to extend lifespan across species (Hansen et al. 2007; Pan et al. 2007; Kapahi et al. 2004; Rogers et al. 2011; Curran and Ruvkun 2007). In mammals, the most commonly cited mechanism for the beneficial effects of tetracyclines is inhibition of matrix metalloproteinase 9 (MMP9) (Golub et al. 1991; Vandenbroucke and Libert 2014; Bruno et al. 2009), originating from observations in diabetes–induced gingivitis (Golub et al. 1983), arthritis (Clay et al. 2023), and cancer (Seo et al. 2013).

The beneficial nonantibiotic effects of tetracyclines have been investigated in a variety of models using different analogs, complicating therapeutic development and obscuring the underlying mechanistic pathway involved in longevity. To clarify the mechanism(s) by which tetracyclines elicit geroprotective effects, we systematically profiled 21 widely used tetracyclines, including known impurities, across *C. elegans* , mammalian cell lines, primary neurons, and human induced pluripotent stem cell (iPSC)‐derived neurons. We discover an atypical tetracycline, 4‐epiminocycline, that is neuroprotective and attenuates translation in the brain when administered to mice, revealing that pharmacological reduction of protein synthesis is sufficient to promote longevity independently of ISR activation. Together, these findings suggest that translation attenuation is a druggable mechanism underlying the geroprotective effects of tetracyclines.

## 1 Results

### 1.1 Tetracyclines Extend the Lifespan of Adult *C. elegans* Independent of Antibiotic Activity

**FIGURE 1:**
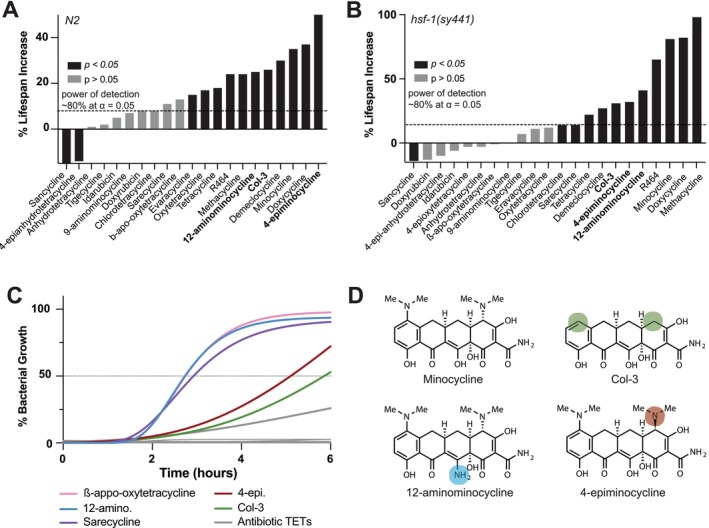
Antibiotic activity is not required for tetracycline‐induced geroprotection. (A) Bar graphs show % change in lifespan for N2 *C. elegans* treated with the indicated tetracycline starting on day 1 of adulthood. (B) Bar graphs show % change in lifespan for *hsf‐1(sy441)* mutants treated with the indicated tetracycline starting on day 1 of adulthood. (C) Bacterial growth as a function of time for *E. coli* (OP50) in the presence of 100 μM of each tetracycline. Tetracyclines that allowed more than 50% growth were considered reduced‐antibiotic, while tetracyclines that had the same growth curve as DMSO were considered nonantibiotic. DMSO is omitted as it is identical to that of β‐appo‐oxytetracyline. DMSO and kanamycin were used as negative and positive controls, respectively. Total of three independent experiments. (D) Chemical structures of minocycline and nonantibiotic tetracyclines that extend lifespan in both N2 and *hsf‐1(sy441)* animals. Colored circles highlight structural changes relative to minocycline. For lifespan screens in A and B, compounds were tested using at least three doses, and the dose with the greatest % increase is shown. Bold indicates compounds active in both strains. Significance was determined by the log‐rank test. Black bars: *p* < 0.05; gray bars: *p* > 0.05. *N* > 50 animals per treatment. The dotted line indicates the estimated power of detection. We expect to identify 80% of all tetracyclines that extend lifespan by 15% or more at an *α* = 0.05. See Supporting Information for more details.

We generated a library of the most widely studied tetracyclines, including several synthesis impurities and degradation products. To evaluate the geroprotective properties of tetracyclines, we determined their ability to extend the lifespan of adult wild‐type *C. elegans* (N2). We identified 11 tetracyclines that increase lifespan (Figure 1A; Table S1). Five additional tetracyclines showed a tendency to extend lifespan but did not reach significance. These may be true negatives or their effect size might fall below our power of detection, which we estimated to be 80% at an *α* = 0.05 for a 15% increase in lifespan (Petrascheck and Miller 2017).

We previously showed that hyper‐translation is a defining phenotype of the HSR‐deficient *hsf‐1(sy441)* mutant (Solis et al. 2018; Clay et al. 2023). Hyper‐translation makes the *hsf‐1(sy441)* mutant especially resistant to many longevity paradigms, including UPR^ER^ activation, reduction of mTOR activity, reduced IIS signaling, dietary restriction, or hormesis (Seo et al. 2013; Steinkraus et al. 2008; Hsu et al. 2003; Kumsta and Hansen 2017; Howard et al. 2016), while sensitizing animals to interventions that lower translation. Thus, we counter‐screened our tetracycline library for their ability to extend the lifespan of *hsf‐1(sy441)* mutants to identify candidate compounds that operate independently of canonical stress response signaling paradigms (Solis et al. 2018; Clay et al. 2023). Eleven tetracyclines extended the lifespan of adult *hsf‐1(sy441)* while two additional tetracyclines showed a tendency but did not reach statistical significance (Figure 1B). There was a strong overlap between groups, with the top 9 hits remaining significant in both backgrounds.

Next, we screened our tetracycline library for antibiotic activity against the Gram‐negative bacterium *E. coli* (Figure 1C). We identified five non‐ or reduced‐antibiotic tetracyclines. These included: 12‐aminominocycline and 4‐epiminocycline (Nelis and Leenheer 1982), two common minocycline impurities, β‐apo‐oxytetracycline, a degradation product of oxytetracycline (Halling‐Sørensen et al. 2002), sarecycline, a narrow‐spectrum antibiotic with low activity against *E. coli* (Zhanel et al. 2018), and Col‐3. The two minocycline impurities, 4‐epiminocycline and 12‐aminominocycline, along with Col‐3, exhibited geroprotective properties while lacking antibiotic activity (Figure 1D). Thus, the lifespan‐extending effect of tetracyclines in eukaryotes is separable from their antibiotic activity.

### 1.2 Tetracyclines Attenuate Eukaryotic Translation

**FIGURE 2:**
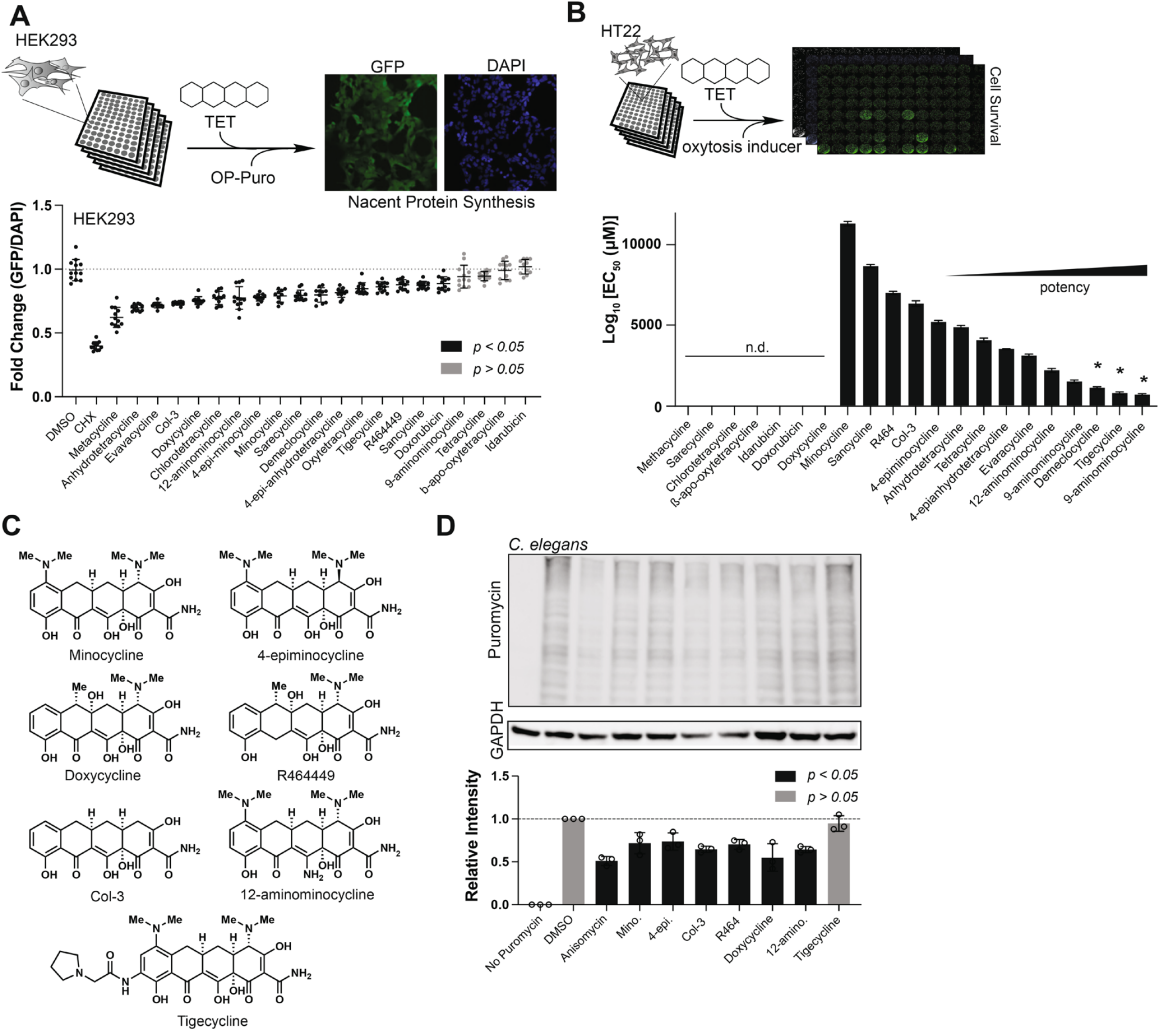
Tetracyclines attenuate translation in eukaryotes. (A) Graph shows fold change in de novo translation in HEK293 cells treated with individual tetracyclines. Each tetracycline was tested at a concentration of 100 μM. Translation was quantified by the OPP incorporation assay, determining the mean OPP incorporation intensity relative to DAPI. Cycloheximide (CHX) serves as a positive control. Total of three independent trials with at least four images quantified per trial. (B) The graph shows Log_10_(EC_50_) for neuroprotection. EC_50_ values were derived from dose–response curves measuring cell viability of hippocampal‐derived HT22 cells subjected to oxytosis/ferroptosis. HT22 cells were pretreated for 4 h with increasing concentrations of tetracyclines, followed by the induction of oxytosis/ferroptosis with glutamate (5 mM) and determination of cell survival 20 h later. Error bars indicate mean ± SD. Data representative of three independent trials. * indicates compounds that were cytotoxic at the highest concentration. (C) Chemical structures of seven diverse tetracyclines, representing an array of structural modifications. (D) The puromycin incorporation assay (SUnSET) was used to monitor the effect of tetracycline treatment on translation in *C. elegans* (100 μM for each compound, except 4‐epiminocycline and 12‐aminominocycline, which were used at 33 and 200 μM, respectively). Immunoblot of protein extracts stained with puromycin antibody reveals reduced puromycin incorporation and hence translation (top). GAPDH is used as a loading control. Quantification of three independent SUnSET trials shows broad translation inhibition of geroprotective tetracyclines. Significance was determined by one‐way ANOVA using Dunnett's multiple comparisons correction, where black represents *p* < 0.05, and gray represents *p* > 0.05.

We previously showed that the geroprotective effect of minocycline is the result of attenuated translation (Solis et al. 2018). We therefore screened our tetracycline library for the reduction of de novo translation using a high‐content imaging approach in HEK293 cells through o‐propargyl puromycin incorporation (OPP) (Liu et al. 2012). The OPP‐labeled proteins are visualized by conjugating a fluorophore using click chemistry (Kolb et al. 2001), and quantified by comparing the green probe signal to the nuclear DAPI signal. Of the 21 tetracyclines, 17 reduced de novo translation in HEK293 cells (Figure 2A). We note that the efficacy of tetracyclines in reducing translation is much weaker than that of the classical translation inhibitor cycloheximide. We therefore refer to the reduction in translation by tetracyclines in eukaryotes as *attenuation*. Our data show that attenuation of translation is a general feature of tetracyclines in eukaryotes.

To support the development of geroneuroprotective compounds (Schubert et al. 2018), we surveyed our tetracycline library in an assay designed to identify compounds that protect hippocampal‐derived HT22 cells from oxytosis/ferroptosis‐mediated cell death. Oxytosis/ferroptosis is a lipid peroxidation‐mediated programmed cell death linked to neurodegenerative diseases. Experimentally, it is induced by high concentrations of glutamate or by using potent and specific inducers such as erastin or RSL3 (Lewerenz et al. 2018; Sarparast et al. 2023). Eleven of the 21 tetracyclines tested were neuroprotective, exhibiting EC_50_ values of 1.5–11 μM (Figure 2B). Of these neuroprotective compounds, three were toxic to the cells at higher concentrations, resulting in an artificially low EC_50_ (denoted by an asterisk).

Minocycline was previously shown using chemical proteomics to target the cytoplasmic ribosome in both *C. elegans* and human cell culture. We hypothesized that tetracyclines that extend lifespan similarly attenuate translation in wild‐type *C. elegans* . We selected five geroprotective tetracyclines, one positive control (minocycline) and one inactive negative control (tigecycline), representing a wide structural diversity of the tetracyclines (Figure 2C), and measured translation attenuation using the same concentration that extends lifespan in *C. elegans* (Arnold et al. 2014; Yan et al. 2025). All six geroprotective tetracyclines attenuated translation (Figure 2D). Tigecycline, while structurally similar, does not extend lifespan and does not reduce translation. Together, these data support translation attenuation as a shared pharmacological activity of lifespan‐extending tetracyclines.

### 1.3 Tetracyclines Elicit Ferroptotic Neuroprotection by ISR‐Dependent and Independent Attenuation of Translation

We next set out to characterize the mechanisms by which tetracyclines attenuate translation. Tetracyclines, like doxycycline, that target the mitochondrial ribosome activate the ISR via the phosphorylation of eIF2α to attenuate cytosolic translation, and activate ATF4 (Mottis et al. 2022; Perry et al. 2021; Molenaars et al. 2020; Ronayne et al. 2023). Alternatively, tetracyclines that target the cytosolic ribosome directly attenuate translation, independently of the ISR (Solis et al. 2018). These two pathways can be distinguished by co‐treatment with the ISR Inhibitor ISRIB (Sidrauski et al. 2013; Zyryanova et al. 2021), which prevents the activation of the ISR through increasing activity of eIF2B, thereby bypassing p‐eIF2α induced translation inhibition.

To determine if the tetracycline‐mediated attenuation of translation is prevented by co‐treatment with ISRIB, we repeated the OPP‐based translation assay in HT22 cells in the presence or absence of ISRIB. We used the same set of tetracyclines as in the *C. elegans* translation assay to simultaneously examine evolutionary conservation. As a positive control for an ISR‐*dependent* inhibition of translation, we included thapsigargin (Sidrauski et al. 2015). As a positive control for the ISR‐*independent* inhibition of translation, we included the classical elongation inhibitor cycloheximide (CHX) (Schneider‐Poetsch et al. 2010).

**FIGURE 3:**
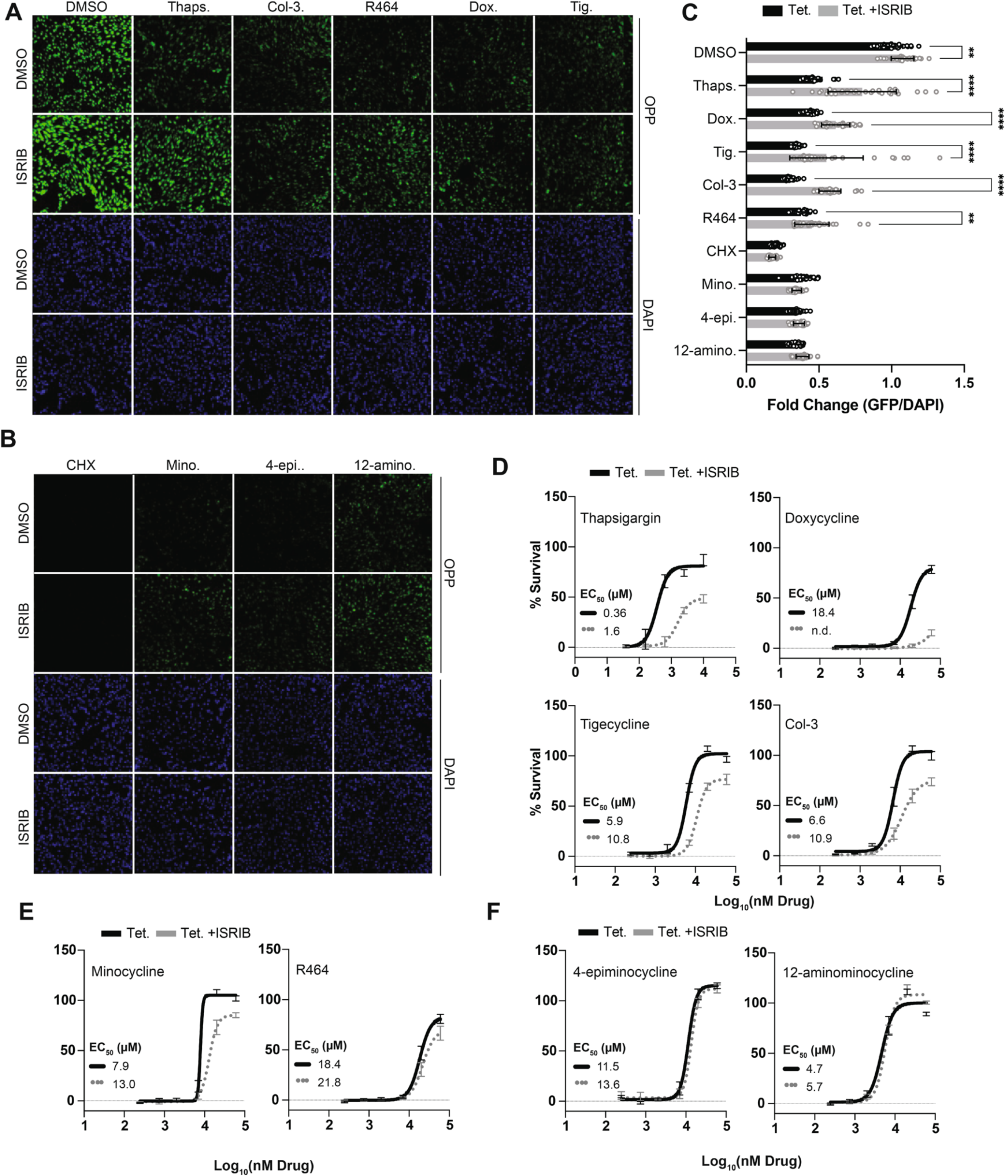
Tetracyclines protect neurons by ISR‐dependent and ISR‐independent attenuation of translation. (A) Representative fluorescent micrographs of HT22 cells stained for OPP incorporation as a readout for de novo translation. Translation is attenuated by ISR‐dependent tetracyclines and restored by co‐treatment with ISRIB. Thapsigargin (Thaps., 1 μM) is included as a control for an ISR‐dependent translation inhibitor. Tetracyclines were screened at 100 μM. (B) Same fluorescent micrographs as in A, but for ISR‐independent tetracyclines whose translation attenuation is unaffected by ISRIB co‐treatment. Cycloheximide (CHX, 500 nM) is included as a control for an ISR‐independent translation inhibitor. (C) Quantification of the OPP incorporation intensity relative to the DAPI signals shown in A and B. Several tetracyclines attenuate translation independently of the ISR (black vs. gray bars). Significance was determined by two‐way ANOVA with Šídák multiple comparisons test. **p < 0.01 and ****p < 0.0001. (D) Graphs show the % survival of HT22 cells as a function of tetracycline dose. The % survival is calculated relative to non‐glutamate‐treated HT22 control cells (100%). HT22 cells were pretreated with ISR‐dependent tetracyclines, alone or in combination with ISRIB, followed by the induction of oxytosis/ferroptosis. Thapsigargin (Thaps.) is used as a positive, ISR‐dependent control. (E) Same as D, but for partially ISR‐dependent tetracyclines. (F) Same as D, but for ISR‐independent tetracyclines. For all figures: Cells were treated for 2 h with ISRIB (300 nM, gray bar, or dotted line) or vehicle control (DMSO, black bar/line) prior to co‐incubation with the indicated tetracycline or control compound. Error bars indicate mean ± SD from three independent trials.

These experiments revealed the existence of two tetracycline classes, ISR‐dependent (Figure 3A,C) and ISR‐independent tetracyclines (Figure 3B,C). The ISR‐dependent tetracyclines, such as doxycycline, were previously shown to act on the mitochondrial ribosome (Mottis et al. 2022; Ronayne et al. 2023). In contrast, the ISR‐independent tetracyclines, such as minocycline, were shown to directly bind to the cytoplasmic ribosome to lower translation (Solis et al. 2018; Mortison et al. 2018). The ability of doxycycline to attenuate translation can be blocked by ISRIB, allowing us to directly test whether the neuroprotection depends on the attenuation of translation. Co‐treatment of doxycycline‐treated cells with ISRIB restored translation and abolished the neuroprotective effect, indicating that attenuation of translation is necessary for neuroprotection.

Based on the data above, we classified doxycycline, Col‐3, and tigecycline as ISR‐dependent (Figure 3D); minocycline and R464 as intermediate (Figure 3E); and 4‐epiminocycline and 12‐aminominocycline as ISR‐independent (Figure 3F). Doxycycline is the clearest ISR‐dependent representative, as ISRIB co‐treatment reduced its neuroprotective properties the most. 4‐epiminocycline and 12‐aminominocycline were the clearest ISR‐independent representatives and were entirely resistant to ISRIB co‐treatment (Figure 3D,F). However, ISRIB co‐treatment did not fully abolish effects on translation or neuroprotection, even for doxycycline, suggesting that all tetracyclines directly attenuate translation to some degree and that parallel ISR activation occurs in some tetracyclines. Based on our antibiotic data and the proposition that mitochondria are ancestrally linked to bacteria and therefore share antibiotic binding sites, we propose that modification in the central pharmacophore important to antibiotic activity (e.g., C11–12 or C4) tends to result in poor antibiotic, but geroneuroprotective, ISR‐independent tetracyclines. Since this structural pattern diverges from historical tetracycline drug discovery and development, we refer to these molecules as *atypical* tetracyclines.

### 1.4 MMP9 Inhibition Is Not Required for Tetracycline‐Induced Neuroprotection

Tetracycline neuroprotection is often attributed to MMP9 inhibition via Zn^2+^ chelation (Lindeman et al. 2009; Rudek et al. 2001; Dezube et al. 2006; Chang et al. 2017), but two analogs we identified (12‐aminominocycline and R464) lack the C11–C12 β‐diketone structure required for chelation (Figure S1A) (Hidalgo and Eckhardt 2001). We therefore tested whether MMP9 inhibition was responsible for neuroprotection. As expected, they failed to chelate Zn^2+^, and even classical tetracyclines did so only weakly, with millimolar IC_50_ values (Figure S1B). In a recombinant MMP9 assay, 5 of 8 tetracyclines inhibited the enzyme at 100 μM (Figure S1C), and dose response curves showed that 12‐aminominocycline and 4‐epiminocycline did not inhibit MMP9 despite being neuroprotective (Figure S1D). Thus, tetracycline–induced neuroprotection can be uncoupled from MMP9 inhibition.

### 1.5 Tetracyclines Extend Lifespan by ISR‐Dependent and Independent Mechanisms in *C. elegans*

We next asked if distinguishing tetracyclines as ISR‐dependent and ISR‐independent also applies to their geroprotective effects. Consistent with this classification, doxycycline is known to inhibit the mitochondrial ribosome, triggering the mitochondrial unfolded protein response (UPR^mt^), which in turn activates the ISR to attenuate translation and upregulate ATF‐4 to extend the lifespan of *C. elegans* (Borankova et al. 2023; Lima et al. 2022; Nargund et al. 2012). Conversely, atypical ISR‐independent tetracyclines are expected to extend lifespan independently of the ISR or of any stress response, such as the heat shock response (HSR) or the UPR^mt^, as stress response signaling converges on the ISR (Derisbourg, Hartman, and Denzel 2021).

**FIGURE 4:**
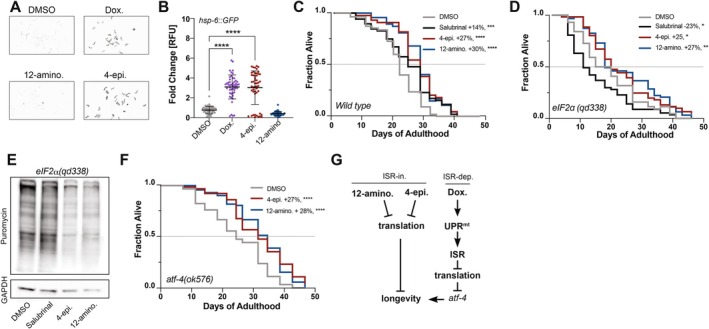
4‐Epiminocycline and 12‐aminominocycline extend lifespan independently of the ISR. (A) Representative fluorescent images of 50 randomly selected *hsp‐6p::GFP* UPR^mt^ reporter animals treated with the indicated tetracycline. Images were taken in parallel following measurement in B. Images were inverted for clarity. (B) Scatter plot shows the fold induction relative to DMSO of the *hsp‐6::GFP* UPR^mt^ reporter in response to 12‐h tetracycline treatment initiated at the L2 stage. Significance was determined by one‐way ANOVA with Dunnett's multiple comparisons, where ****p < 0.0001. Error bars indicate mean ± SD of three independent trials. Significance was determined by two‐way ANOVA with Šídák multiple comparisons test. ****p < 0.0001. (C) Survival plot of wild‐type (N2) animals treated with tetracyclines or the ISR‐dependent translation inhibitor salubrinal. Tetracycline and salubrinal treatment extend the lifespan of N2. Significance was determined by a Log‐Rank test. *p < 0.05, **p < 0.01, ***p < 0.001, and ****p < 0.0001. (D) Survival plot of ISR‐deficient *eIF2*⍺*(qd338)* mutants treated with tetracyclines or salubrinal. Only tetracyclines, but not salubrinal treatment, extend the lifespan of *eIF2*⍺*(qd338)* mutants. For similar data on *eIF2*⍺*(rog3)*, see Figure S2D. Significance was determined by a Log‐Rank test. *p < 0.05, **p < 0.01, ***p < 0.001, and ****p < 0.0001. (E) A puromycin incorporation assay (SUnSET) was used to monitor the effect of tetracycline or salubrinal treatment on translation in the ISR‐deficient *eIF2⍺(qd338)* mutant. Only tetracyclines, but not salubrinal treatment, reduce translation in the ISR‐deficient *eIF2⍺(qd338)* mutant. GAPDH was used as a loading control. For quantification, see Figure S2E. (F) Survival plot of the partially ISR‐deficient *atf‐4(ok576)* mutants treated with tetracyclines. Tetracyclines extend lifespan mostly independent of ATF‐4. For corresponding survival data on doxycycline, see Figure S2F,G. Significance was determined by a Log‐Rank test. *p < 0.05, **p < 0.01, ***p < 0.001, and ****p < 0.0001. (G) Schematic outlining distinguishing features between tetracyclines. 12‐Aminominocycline and 4‐epiminocycline directly inhibit translation to extend lifespan, while ISR‐dependent tetracyclines, as exemplified by doxycycline, activate the mitochondrial UPR to inhibit translation and subsequently extend lifespan in an *atf‐4* dependent manner. The significance of all survival data was determined by the log‐rank test. In B–F, the final concentration of 100 μM was used for doxycycline and salubrinal, 33 μM for 4‐epiminocycline, and 200 μM for 12‐aminominocycline.

Since we found several tetracyclines to extend the lifespan of the *hsf‐1(sy441)* mutant, we evaluated the induction of the *hsp‐16.2::GFP* heat shock reporter after treatment with two ISR‐dependent and two ISR‐independent tetracyclines, followed by a heat shock. We found both to inhibit rather than activate the HSR (Figure S2A–C). To probe the involvement of the UPR^mt^, we evaluated the ability of the most typical ISR‐dependent tetracycline, doxycycline, and the ISR‐independent atypical tetracyclines, 4‐epiminocycline and 12‐aminominocycline, to induce the UPR^mt^ reporter *hsp‐6::GFP* (Yoneda et al. 2004). Consistent with their distinction, doxycycline induced the UPR^mt^ while 12‐aminominocycline did not. Surprisingly, 4‐epiminocycline induced the UPR^mt^ (Figure 4A,B).

The induction of the UPR^mt^ by 4‐epiminocycline suggested it might act ISR‐dependently in *C. elegans* but ISR‐independently in mammals. To classify 4‐epiminocycline, we determined its dependence on the ISR to extend lifespan. We measured the ability of 4‐epiminocycline and 12‐aminominocycline to extend the lifespan of the ISR‐deficient *eIF2*⍺*(rog3)* (Rollins et al. 2017) and *eIF2*⍺*(qd338)* (Kulalert et al. 2017) strains. These strains carry phospho‐deficient S49A and S49P mutations that prevent *eIF2*⍺ phosphorylation and thus ISR activation. As a control, we included salubrinal, an ISR‐dependent translation initiation inhibitor that extends the lifespan of *C. elegans* (Clay et al. 2023; Boyce et al. 2005). 4‐epiminocycline and 12‐aminominocycline treatment extended the lifespan of ISR‐deficient strains (Figure 4C,D, Figure S2D) and attenuated translation in the *eIF2α(qd338)* mutant (Figure 4E, Figure S2E), while salubrinal treatment did neither. We conclude that 4‐epiminocycline and 12‐aminominocycline extend lifespan and attenuate translation independently of the ISR. Even though 4‐epiminocycline activated the UPR^mt^, its lifespan extension does not depend on it.

To further corroborate 4‐epiminocycline and 12‐aminominocycline as ISR‐independent and doxycycline as ISR‐dependent, we determined the requirement of ATF‐4, an ISR transcription factor downstream of *eIF2α* phosphorylation. Both 4‐epiminocycline and 12‐aminominocycline extended the lifespan of *atf‐4(ok576)* mutants (Figure 4F), while the effect of doxycycline was severely blunted (Figure S2F,G). Thus, the mechanistic classification of the atypical tetracyclines 4‐epiminocycline and 12‐aminominocycline as ISR‐independent and of doxycycline as ISR‐dependent applies to geroprotection as well (Figure 4G).

### 1.6 Atypical Tetracyclines Are Neuroprotective and Attenuate Translation in Human Neurons

**FIGURE 5:**
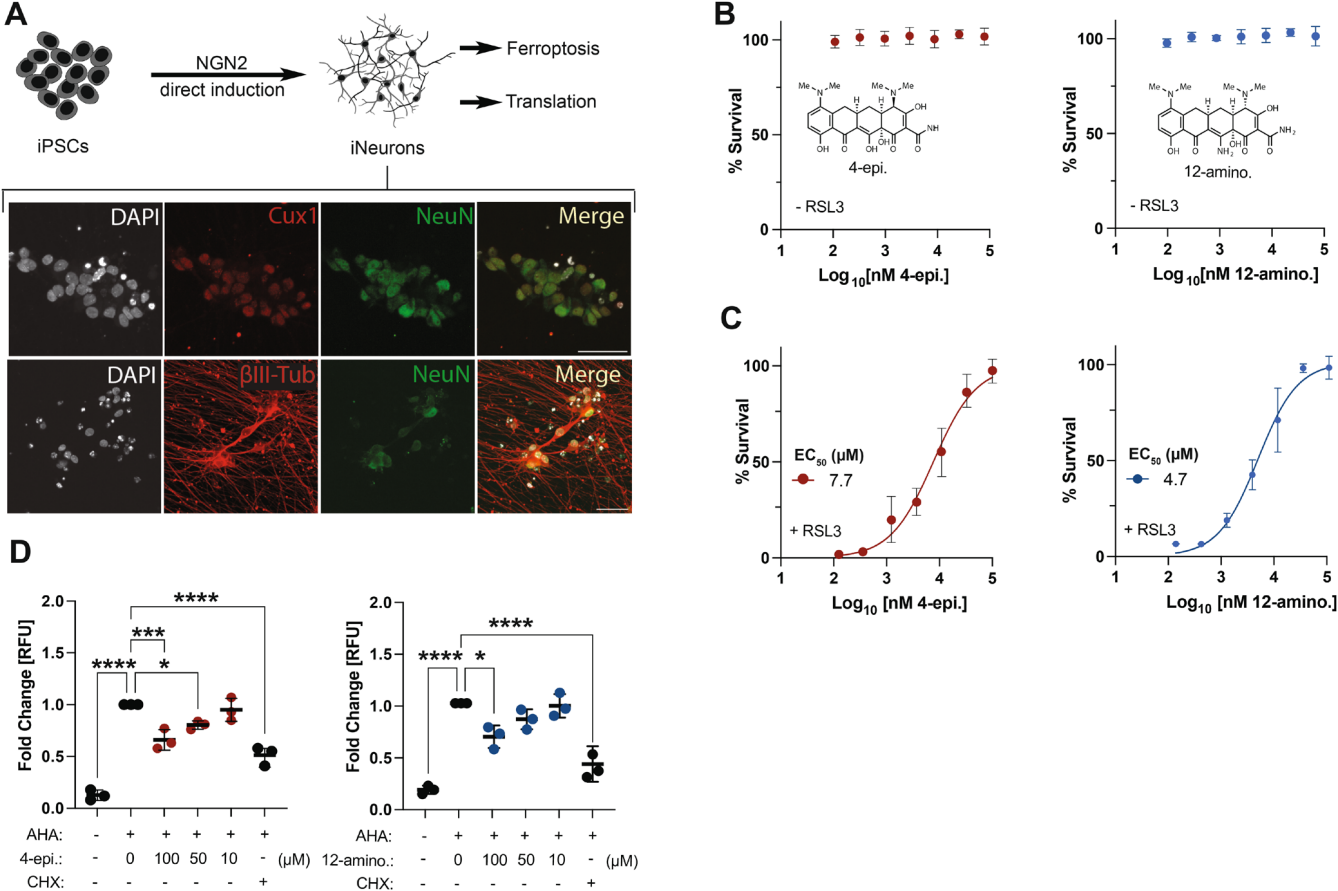
Atypical tetracyclines protect human neurons from ferroptosis and inhibit protein synthesis. (A) Experimental scheme to measure translation and neuroprotection in human induced neurons (iNs). (Top) Cropped representative confocal images of immunofluorescent labeling of generated iNs. Nuclei (DAPI, white), cortical marker CUX1 (Cux1, red), and neuronal marker (NeuN, green) stains are shown. (Bottom) Validation of differentiation by staining for nuclei (DAPI, white), the pan neuronal marker TUBB3 (βIII‐Tub, red), and the neuronal marker (NeuN, green). (B) No cytotoxicity was detected in iNeurons 48 h after either atypical tetracycline addition. (C) Survival of iNeurons as a function of 4‐epiminocycline (red) and 12‐aminominocycline (blue) concentration after ferroptosis induction by RSL3 (2 μM). (D) Quantification of de novo translation in iNeurons treated with increasing concentration of indicated atypical tetracycline using FUNCAT. iNeurons were co‐incubated with (AHA, 8 mM) and increasing concentrations of compound for 4 h, followed by lysis and bioconjugation of an Alexa‐488 alkyne to the incorporated AHA by a click reaction. The protein synthesis inhibitor cycloheximide (CHX, 250 nM) was used as a positive control. Significance was determined by one‐way ANOVA with Dunnett's multiple comparisons, where *p < 0.05, ***p < 0.001, and ****p < 0.0001. All error bars indicate mean ± SD from 3 to 6 independent trials or animals. Cell survival was measured using Cell Titer Glo 20 h after treatment in all experiments.

We then tested the ability of 4‐epiminocycline and 12‐aminominocycline to protect human neurons from oxytosis/ferroptosis. We used human (iPSC) line HE463#7 to differentiate into neurons (iNeurons) (Verduzco Espinoza et al. 2025). We confirmed the neuronal identity of iNeurons by expression of the neuronal markers NeuN, Cux1, and TUBB3 (Figure 5A). Importantly, both atypical tetracyclines were nontoxic to iNeurons (Figure 5B). We then pretreated iNeurons with 4‐epiminocycline or 12‐aminominocycline and induced oxytosis/ferroptosis with RSL3. Both 4‐epiminocycline and 12‐aminominocycline dose‐dependently increased the survival of human iNeurons with EC_50_ values of 7.7 and 4.7 μM, respectively (Figure 5C). We then measured translation using FUNCAT and found a dose‐dependent attenuation of translation by a maximum of 40% at the highest concentration (Figure 5D). Together, we conclude that atypical nonantibiotic tetracyclines are neuroprotective in human neurons, providing an interesting new strategy for therapeutic development.

### 1.7 4‐Epiminocycline Is Brain Penetrant and Attenuates Activity‐Induced Protein Synthesis In Vivo

Previous studies have shown that reducing translation by inhibiting the ISR can improve age‐related cognitive decline in mice (Krukowski et al. 2020) and extend lifespan in *C. elegans* (Derisbourg, Wester, et al. 2021). ISR‐independent, nonantibiotic neuroprotective tetracyclines would make attractive therapeutics for treating aging and age‐related diseases in cases where continued ISR activation may be problematic.

Although 12‐aminominocycline showed a cleaner profile than 4‐epiminocycline, including no detectable MMP9 activity and no UPRmt activation in *C. elegans* , we found that 12‐aminominocycline was less stable in solution (Figure S3A). Using LC–MS, we found that 12‐aminominocycline rapidly degraded over 72 h, with only trace parent compound remaining after 7 days (Figure S3B). Conversely, 4‐epiminocycline was intact over the measurement period. We therefore focused on 4‐epiminocycline as a proof‐of‐principle tool compound for in vivo studies, testing whether translation attenuation in the mammalian brain is feasible.

**FIGURE 6:**
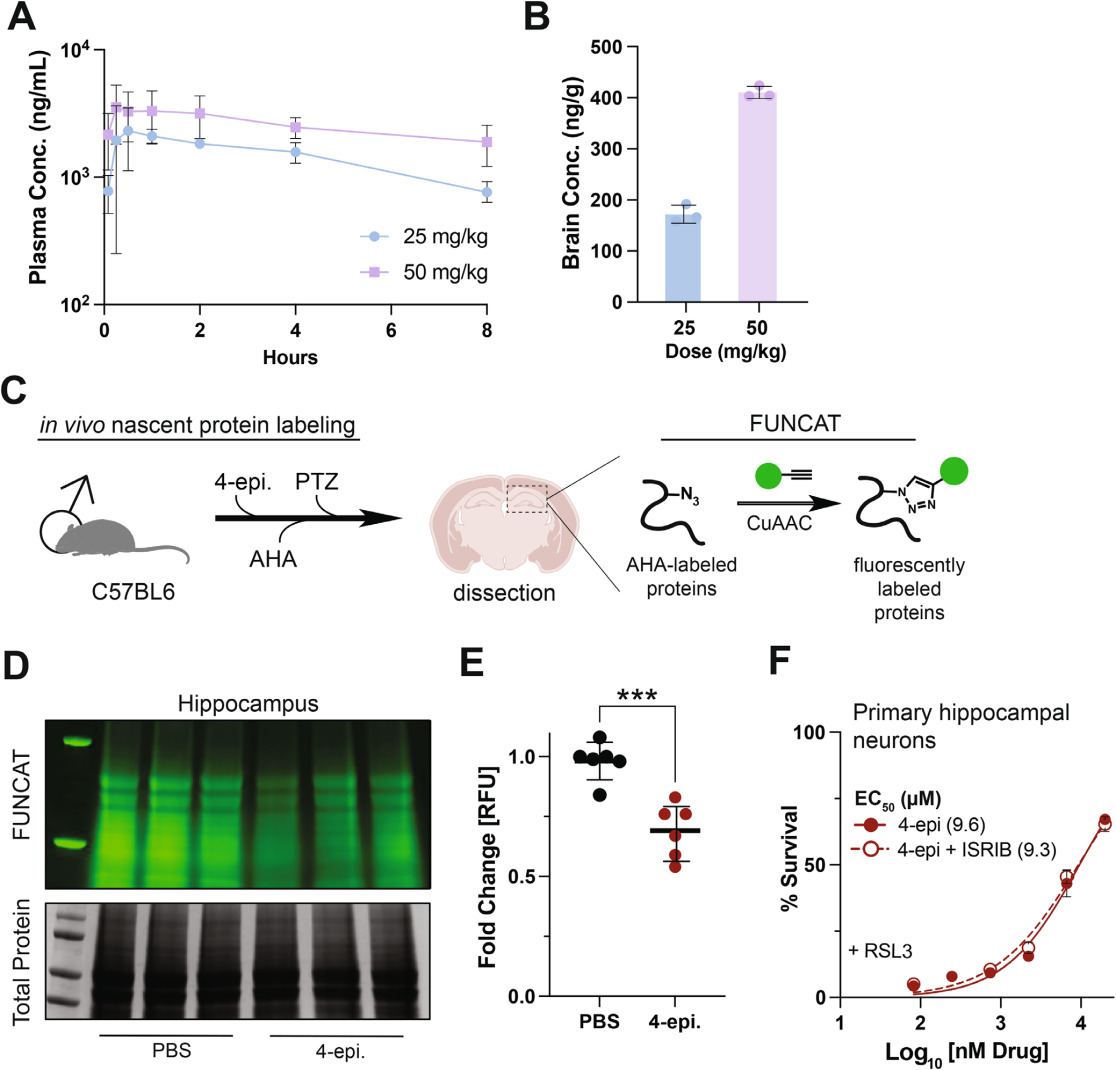
4‐Epiminocycline is brain penetrant and attenuates activity‐induced protein synthesis in vivo. (A) Plasma concentrations of 4‐epiminocycline over 8 h following a single intraperitoneal injection (i.p.) of male Sprague–Dawley rats. (B) Brain concentrations of 4‐epiminocycline after 8 h following a single intraperitoneal injection of male Sprague–Dawley rats. (C) Experimental scheme to measure de novo translation by FUNCAT in the brains of male C57BL/6 mice. Mice were treated with 4‐epiminocycline in drinking water for 3 days, followed on day 4 by 4‐epiminocycline i.p. injection (100 mg/kg), AHA injection (50 mg/kg), and PTZ injection (50 mg/kg) 30 min later to induce neuronal protein synthesis. The hippocampus was micro‐dissected and lysed to fluorescently label newly synthesized proteins that incorporated AHA by click chemistry. (D) Fluorescent‐scan (top) and Coomassie blue stain (bottom) gel. In‐gel fluorescence detects de novo translation by the amount of incorporated fluorescently labeled AHA (top) relative to total protein (bottom). 4‐Epiminocycline reduced de novo translation in the hippocampus of mice. (E) Quantification of FUNCAT experiments shows 4‐epiminocycline reduces de novo translation by 25%. Significance was determined by a two‐tailed Student's *t*‐test, where ***p < 0.001 and *N* = 6. (F) Survival of primary hippocampal neurons as a function of 4‐epiminocycline concentration after ferroptosis induction by RSL3 (300 nM). Co‐treatment with ISRIB (300 nM) does not abolish the neuroprotective effect of 4‐epiminocycline, confirming ISR‐independence.

An initial pharmacokinetic (PK) evaluation of 4‐epiminocycline in rats established significant exposure in both the periphery and CNS. A single 50 mg/kg dose (i.p.) resulted in plasma and brain concentrations of 1887 ng/mL and 410 ng/g, respectively, while a 25 mg/kg dose resulted in plasma and brain concentrations of 764 ng/mL and 172 ng/g, respectively (Figure 6A), 8 h postinjection (Figure 6B). Thus, 4‐epiminocycline is brain‐penetrant and has a low clearance in both plasma and the CNS.

We then measured the ability of 4‐epiminocycline to attenuate translation in the mouse brain in vivo by Fluorescent Noncanonical Amino Acid Tagging (FUNCAT). FUNCAT measures the incorporation of azidohomoalanine (AHA) into newly synthesized proteins and visualization by bioconjugation of a TAMRA‐alkyne using click chemistry (Figure 6C; Xie et al. 2025). We treated male C57BL/6 mice with 4‐epiminocycline in the drinking water for 3 days, followed by an intraperitoneal (i.p.) co‐injection with 4‐epiminocycline plus AHA to label newly synthesized proteins. Since neuronal activity promotes translation (Xie et al. 2025), we injected the mice with pentylenetetrazol (PTZ) 30 min after the AHA injection to evaluate the ability of 4‐epiminocycline to inhibit the synthesis of nascent proteins. Two hours post‐PTZ injection, the mice were sacrificed, and hippocampal proteins were extracted to quantify translation. Treatment with 4‐epiminocycline significantly attenuated hippocampal translation by ~25% (Figure 6D,E), indicating that 4‐epiminocycline attenuates activity‐induced translation in vivo to the same degree as observed in all other paradigms.

We further determined if 4‐epiminocycline affects mitochondria in vivo. It has been shown that doxycycline disrupts the mitonuclear translational balance, which can be visualized by the disruption of the stoichiometric ratio between nuclear‐encoded electron transport chain components (ETC), such as ATP5A, and mitochondrial‐encoded ETC components like MT‐CO1 (Houtkooper et al. 2013). We did not observe any selective suppression of the mitochondrial‐encoded MT‐CO1, indicating that 4‐epiminocycline does not inhibit mitochondrial translation (Figure S3C,D). It is essential to note that we normalized each lane by protein amount and the loading control actin. This method obfuscates a cytosolic ribosome‐specific inhibitor since both Actin and ATP5A are equally reduced. Taken together, these data show that 4‐epiminocycline does not target the mitochondrial ribosome.

We then pretreated primary mouse neurons with 4‐epiminocycline and measured survival following induction of oxytosis/ferroptosis (Yang and Stockwell 2008) in the presence and absence of ISRIB. As expected for an ISR‐independent tetracycline, the dose‐dependent improvement of survival of 4‐epiminocycline‐treated neurons was unaffected by ISRIB co‐treatment (Figure 6F). ISRIB‐mediated restoration of translation following ISR‐dependent translation inhibition was independently confirmed in primary neurons. Treatment of the ISR‐dependent translation inhibitor tunicamycin reduced protein synthesis, and this effect was rescued by ISRIB co‐treatment using the same timepoints and concentrations as in Figure 6F (Figure S3E) (Sidrauski et al. 2015; Szaruga et al. 2023). The treatment and testing for the ability to protect from oxytosis/ferroptosis of primary neurons does not require long‐term stability, allowing us to evaluate 12‐aminominocycline. 12‐aminominocycline protected primary hippocampal neurons, confirming that atypical tetracyclines are neuroprotective across a variety of models we employed (Figure S3F).

## 2 Discussion

The neuroprotective, anti‐inflammatory, and geroprotective effects of tetracyclines are well‐documented in the preclinical and clinical trial literature, including for many age‐associated diseases. While the literature on the potential therapeutic effects of different tetracyclines is extensive, no systematic study has been conducted to compare and evaluate the underlying molecular mechanisms. Here, we profiled the most widely used tetracyclines and their impurities to characterize their geroprotective and neuroprotective activities and mechanisms of action. We show that translation attenuation is a shared mechanism underlying the geroprotective and neuroprotective effects of all tetracyclines. Despite the shared effect on translation, tetracyclines can be distinguished by their dependence on the ISR into typical (ISR‐dependent) and atypical (ISR‐independent) tetracyclines. The nonantibiotic tetracyclines identified here do not require ISR activation to attenuate translation. They are referred to as atypical tetracyclines based on their structural dissimilarity to historical antibiotic drug discovery. These compounds harbor changes in the so‐called “non‐modifiable region” of the tetracyclines.

Our data establish: (i) Modifications in the C4 or C11,12 positions of tetracyclines weaken their antibiotic and MMP9 inhibitory activity and uncouple their neuroprotective and geroprotective effects from antibiotic activity. (ii) Instead, the neuroprotective and geroprotective effects of tetracyclines are the result of attenuation of translation. (iii) The mechanism of action by which tetracyclines attenuate translation classifies them into ISR‐dependent and ISR‐independent tetracyclines (Figures 3 and 4). Translation inhibition (and downstream neuroprotection) by ISR‐dependent tetracyclines like doxycycline can be blocked by ISRIB, while ISR‐independent tetracyclines cannot. (iv) Atypical tetracyclines like 4‐epiminocycline show reduced antibiotic activity, are brain penetrant, and protect both mouse and human neurons from oxytosis/ferroptosis (Figures 5 and 6).

We also uncoupled the neuroprotective effects from MMP9 inhibition by showing that structurally related minocycline analogs fail to inhibit MMP9 but remain neuroprotective (Figure S1). This evidence challenges the pervasive assertion that MMP9 inhibition is the causal mechanism underlying tetracycline neuroprotection, consistent with past failures of specific MMP inhibitors to recapitulate the properties of tetracyclines. Although we exclude a direct engagement of atypical tetracyclines with MMP9, it is still possible that they reduce the protein levels of MMP9 and that this reduction indirectly contributes to the protective effect.

We then focused on seven structurally diverse tetracyclines to evaluate the attenuation of translation as the underlying mechanism for neuroprotection (Solis et al. 2018; Houtkooper et al. 2013; Ye et al. 2014; Lombard et al. 2020). All seven neuroprotective tetracyclines lower protein synthesis but do so through ISR‐dependent and ISR‐independent mechanisms (Figure 3). The ISR dependency of doxycycline enabled us to demonstrate that the attenuation of translation directly causes ferroptotic neuroprotection, since the ISR inhibitor ISRIB restored translation and abolished neuroprotection during doxycycline treatment, revealing a causal relationship (Figure 3). The ISR‐based classification of tetracyclines also applies to their effect on longevity (Anisimova et al. 2018). Doxycycline induces the UPR^mt^ and ISR and requires ATF‐4 to extend lifespan (Mottis et al. 2022; Shao et al. 2025; Perry et al. 2021; Molenaars et al. 2020; Ronayne et al. 2023; Vendramin et al. 2021; Borankova et al. 2023). Conversely, 12‐aminominocycline and 4‐epiminocycline do not require the ISR nor ATF‐4 (Figure 4). However, our data also suggest that many tetracyclines fall in between the ISR‐dependent/independent extremes and are partially ISR‐dependent.

Many antibiotics that inhibit bacterial ribosomes also inhibit mitochondrial ribosomes by identical mechanisms (Marks et al. 2024; Almeida et al. 2021). Cryo‐EM structures of tigecycline bound to the mitoribosome showed binding in the peptidyl transferase center that overlapped with the binding site for structurally related tetracyclines in bacterial ribosomes (Shao et al. 2025). A recent screen aimed at identifying mitochondrial‐targeting tetracyclines with limited antibiotic activity identified 9‐t‐butyl‐doxycycline as a therapeutically developable compound for influenza (Kapahi et al. 2004). Notably, none of the 18 tetracyclines derived from the minocycline scaffold activate the UPR^mt^, confirming that doxycycline‐related tetracyclines tend to target mitochondrial ribosomes, whereas minocycline‐related tetracyclines tend to target cytosolic ribosomes (Solis et al. 2018). Thus, we proposed that the tetracyclines represent a molecular scaffold whose neuroprotective and geroprotective properties can be tuned to be ISR‐dependent or independent by either targeting the mitochondrial or cytosolic ribosome. This proposition remains to be tested and will require extensive effort to simultaneously monitor subcellular compartmental translation at scale. We cursorily examined this question by probing ETC components synthesized by the cytosolic or mitochondrial ribosome in the hippocampus of 4‐epiminocycline‐treated mice and did not observe any differences (Figure S3C). Probing both the subcellular partitioning and binding affinity of each tetracycline to the cytosolic or mitochondrial ribosome will be a great resource to the future development of geroprotective tetracycline‐based translation attenuators.

Here we report the discovery of an atypical tetracycline, 4‐epiminocycline, as a geroprotective compound. 4‐epiminocycline is the epimerization product of minocycline and only differs in the stereochemistry of the C4 position, resulting in low antibiotic activity, while remaining brain penetrant and neuroprotective (Figures 1 and 5). Importantly, 4‐epiminocycline was neuroprotective in HT22 cells, as well as primary mouse neurons and human iNeurons, independent of the ISR (Figure 5E,H; Mottis et al. 2022). 4‐epiminocycline attenuated translation in the hippocampus of mice, the whole body of *C. elegans* , and in human iNeurons by ~25% (Figures 5 and 6; Greenberger 1967).

Our study includes several limitations. In vivo, we utilized PTZ to stimulate neuronal activity and downstream protein synthesis, thereby mimicking an excitotoxic challenge to neuronal homeostasis. We therefore did not directly measure basal translation in the absence of PTZ, and so it remains to be determined how 4‐epiminocycline affects steady‐state translation. The dosing regimen used to determine brain penetrance differed from the regimen used to measure activity‐induced protein synthesis. The PK experiment was designed to establish brain exposure after acute dosing, whereas the FUNCAT experiment used a longer treatment paradigm, including administration in the drinking water, to maintain compound exposure during the labeling window. For the diastereomer minocycline, drinking‐water administration produces plasma levels of approximately 7 μM, which is comparable to circulating levels in human patients of 2–11 μM (Clark‐Walker and Linnane 1966; Chatzispyrou et al. 2015). We note that 4‐epiminocycline extended lifespan at 33 μM but shortened lifespan at higher concentrations (Table S1), indicating a concentration‐dependent therapeutic window. This window mirrors our prior observations with minocycline, where high concentrations produced detrimental effects in *C. elegans* that were not explained by excessive translation attenuation (Solis et al. 2018). We speculate that this off‐target is specific to *C. elegans* , as high concentrations of 4‐epiminocycline were not deleterious to human neurons (Figure 5B). Thus, while partial translation attenuation may be beneficial, higher concentrations of some tetracyclines may engage additional toxic targets.

Minocycline, doxycycline, and other tetracyclines have been tested for their beneficial effects across various indications related to aging but unrelated to their antibiotic activity (Boyce et al. 2005). However, the results in human clinical trials have been mixed (Howard et al. 2020). One of the critical limitations mentioned in many studies is the antibiotic activity, which is both dose‐limiting and a liability for chronic use due to associated gastrointestinal side effects and the important role of the microbiome in aging (Howard et al. 2020; Haïk et al. 2014; Partridge et al. 2020; Ghosh et al. 2022). Several recent studies, including this one, have demonstrated that the beneficial preclinical effects can be uncoupled from the antibiotic activity, as illustrated by 4‐epiminocycline. In addition, the surprising mechanistic diversity of tetracyclines has clear implications for the interpretation of past and future clinical trials. The results of both failed and successful trials need to be reconsidered in light of the ISR dependency of individual tetracyclines. Activation of the ISR can have both beneficial and detrimental effects, depending on the disease indication, and thus drive the failure or success of a trial. Similarly, when generating novel nonantibiotic tetracycline derivatives, it will be crucial to consider the ISR dependency of the parent scaffold and to match it in the nonantibiotic derivatives. Finally, our identification of minocycline impurities with superior efficacy and potency may explain at least some of the mixed or disappointing results in clinical studies, as it represents a variable that was poorly controlled for in any study. Pharmacologically active metabolites with higher potency than the parent compound are well‐documented in drug discovery (Fura 2006). Overall, our study suggests that tetracyclines, with their extensive safety profile, offer the possibility to target translation in humans, engaging one of the most potent lifespan‐extending mechanisms.

## 3 Experimental Models

### 3.1 Mouse Studies

All procedures are approved by TSRI IACUC committee. C57BL/6 male mice (Stock #000664) were purchased from Jackson Labs at 9 weeks of age. Mice were group‐housed and maintained on a 12 h light/dark cycle with ad libitum access to food and water.

### 3.2 *C. elegans* Strains

The Bristol strain (N2) was used as the wild‐type strain. In addition, the following worm strains used in this study were obtained from the Caenorhabditis Genetics Center (CGC; Minneapolis, MN, USA): SJ4100 [zcIs13 [*hsp‐6p::GFP*]], CL2070 [dvIs70 [*hsp‐16.2p::GFP + rol‐6(su1006)*]], PS3551 [*hsf‐1(sy441*)], RB790 [*atf‐4(ok576)*], ZD1866 [*eIF2a(qd338)*], ANR165 [*eIF2a(rog3)*]. Strains were backcrossed at least three times prior to experimental analysis.

### 3.3 Cell Culture

HEK293 cells (ATCC CRL‐1573) were directly obtained from ATCC. HT22 neuronal cells were obtained as a gift from Dr. Pamela Maher. Cells were grown in Dulbecco's modified Eagle medium (DMEM, ThermoFisher, Cat#11995065) supplemented with 10% fetal bovine serum (FBS, ThermoFisher, Cat#16000044) and 1% penicillin–streptomycin (P/S, ThermoFisher, Cat#15140‐122). Cells were subcultured every 2 or 3 days at 37°C with 5% CO_2_ in a humidified incubator. Mycoplasma testing was performed every 6 months through TSRI.

Primary neurons were harvested as previously described in Chassefeyre et al. (2021). Briefly, the hippocampi or cortices of male and female C57BL/6 P0‐P1 mouse pups were dissected in Hanks' balanced salt solution (HBSS) (ThermoFisher, Cat#24020117) supplemented with 0.08% d‐glucose (Sigma‐Aldrich, Cat#G6152), 0.17% HEPES (Sigma‐Aldrich, Cat#H7006), and 1% P/S; filter‐sterilized; and adjusted to pH 7.3. Dissected brain tissues were washed twice with cold HBSS and individually incubated at 37°C for 15–20 min in a sterile solution of 45 U of papain (Worthington, Cat#LS003119), 0.01% deoxyribonuclease (DNase) (Sigma‐Aldrich, Cat#D4527), 1 mg of DL‐cysteine (Sigma‐Aldrich, Cat#C9768), 1 mg of bovine serum albumin (BSA) (Sigma‐Aldrich, Cat#A7906), and 25 mg of D‐glucose in phosphate‐buffered saline (PBS) (ThermoFisher, Cat#10010049). Tissues were washed twice with DMEM supplemented with 10% FBS (preheated to 37°C) and disrupted by 10–12 cycles of aspiration through a micropipette tip. Dissociated neurons were then resuspended in warm DMEM supplemented with 10% FBS, counted and plated in either 96‐well plates (Millipore Sigma, Cat#CLS3603) or 10 cm dishes (Genesee Scientific, Cat#25‐202) pretreated with poly‐L‐lysine (50 μg/mL) (Sigma‐Aldrich, Cat#P5899) in borate buffer (1.24 g of boric acid [Thermo Fisher Scientific, Cat#BP168‐1] and 1.90 g of borax [Sigma‐Aldrich, Cat#B9876] in 500 mL of cell culture–grade water, adjusted to pH 8.5, and filter‐sterilized). Plating densities were 2 × 10^4^ cells per well or 7.75 × 10^6^ cells per dish. After 3 h, medium was replaced with Neurobasal‐A media (ThermoFisher, Cat#10888022), supplemented with 2% antioxidant free (ThermoFisher, Cat#10889038) or complete (ThermoFisher, Cat#17504044) B27 and 0.25% GlutaMAX (ThermoFisher, Cat#35050061). Cultures were maintained in culture media at 37°C with 5% CO_2_. Four days after plating (DIV4), 5‐fluoro‐2′‐deoxyuridine (FUDR, Millipore Sigma, Cat#F0503) was added to a final concentration of 5 μM in culture media.

Induced neurons (iNeurons) were previously generated in Verduzco Espinoza et al. (2025). In short, iNeurons were generated from APOE3/E3 (HE463#6) iPSCs, which are available on WiCell in a collection called Topol Lab's Next Gen Cell Lines with the accession number of SCRP2307i. iPSCs were thawed in mTeSR Plus with 10 μM Y‐27632 (Stem Cell Technologies, Cat#72304) and seeded on 6‐well dishes coated with Matrigel. For maintenance, iPSCs were fed every 1–3 days, depending on confluence, and passaged 1–2 times per week as small clusters using 1 μM EDTA (Invitrogen, Cat#15575‐020). 1× P/S was added to all culture media. Neurons were generated from iPSCs via direct induction with NGN2. iPSCs were dissociated with Accutase (Stem Cell Technologies, Cat#7920) and seeded on Matrigel‐coated 6‐well plates at 1.5 × 10^5^ cells per well in mTeSR Plus with 10 μM Y‐27632. The next day (day 0), each well was transduced with 125 μL of tetO‐NGN2 and RTTA lentivirus for 2 h in mTeSR Plus with 10 μM Y‐27632 at 37°C. After 2 h, the viral media was aspirated and replaced with mTeSR Plus with 2 μg/mL doxycycline (Stem Cell Technologies, Cat#72742) to start NGN2 expression. On day 1, cells were fed with half mTeSR plus half Neuronal media (Neurobasal A, 1× B27, NEAA, Glutamax [ThermoFisher, Cat#10888022, 17504044, 350500661, 11140050]) with doxycycline. NGN2‐expressing cells were selected on day 2 by media exchange with doxycycline and puromycin (Gibco, Cat#A11138‐03). On day 3, cells were replated at 6.25 × 10^4^ cells per cm^2^ on plates coated with PDL and Matrigel. 40 nM BRDU was added to select against the remaining dividing iPSCs. Cells underwent a full media change on days 7 and 10 to remove doxycycline and add neuronal differentiation factors (BDNF, GDNF, NT3 at 10 ng/mL, and laminin at 1 μg/mL). Experiments in this manuscript begin on day 14 for FUNCAT and day 24 for ferroptosis cell survival. For the ferroptosis cell survival experiment, cells underwent a half‐media change on days 14 and 17.

### 3.4 Compounds

#### 3.4.1 Tetracyclines

Minocycline (M.P. Biomedicals, Cat#155718), 4‐epiminocycline (Toronto Research Chemicals, Cat#TRC‐E588540), doxycycline (Sigma, Cat#D9891), Col‐3 (Incyclinide, MedChemExpress, Cat#HY‐13648), R464449 (Sigma‐Aldrich, Cat#R464449), 12‐aminominocycline (Toronto Research Chemicals, Cat#TRC‐A618285), tigecycline (LKT Laboratories, Cat#T3324). Other translation inhibitors: Cycloheximide (CHX, EMD Millipore, Cat#239763), tunicamycin (Tm, Cayman Chemical, Cat#11445), thapsagargin (Thaps., MedChemExpress, Cat#HY‐13433). Following the initial hit, 4‐epiminocycline and 12‐aminominocycline were synthesized from minocycline to confirm purity and compound identity.

#### 3.4.2 4‐Epiminocycline

^**1**^ **H NMR** (400 MHz, DMSO‐*d*_6_): *δ* 15.06 (s, 1H), 11.48 (s, 1H), 9.48–9.29 (m, 2H), 7.49–7.40 (m, 1H), 7.38 (s, 1H), 6.85–6.80 (m, 2H), 4.71 (s, 1H), 3.05–2.65 (m, 8H), 2.65–2.750 (m, 2H), 2.20–2.00 (m, 1H), 1.55–1.41 (m, 1H). **LCMS:** calc. for C_23_H_27_N_3_O_7_: 457.18, found: [M + H]^+^ 458.1. **HPLC:** 99.2% (254 nm). **SFC:** 97.7%.

#### 3.4.3 12‐Aminominocycline

^**1**^ **H NMR** (400 MHz, DMSO‐*d*_6_): δ 13.17–13.03 (m, 1H), 10.72–10.13 (m, 1H), 9.69–9.46 (m, 1H), 9.10–8.44 (m, 1H), 7.16 (d, J = 8.8 Hz, 1H), 6.76 (d, J = 8.8 Hz, 1H), 6.46–6.21 (m, 1H), 5.95 (br s, 1H), 3.23 (dd, J = 4.0, 15.2 Hz, 1H), 2.98 (d, J = 1.6 Hz, 1H), 2.93–2.74 (m, 1H), 2.65 (br t, J = 2.4 Hz, 1H), 2.62 (t, J = 2.4 Hz, 1H), 2.57 (s, 6H), 2.47 (s, 4H), 2.39 (br s, 2H), 2.09 (br t, J = 14.4 Hz, 1H), 1.93 (br dd, J = 5.2, 7.6 Hz, 1H), 1.46–1.35 (m, 1H). **LCMS**: calc. for C_22_H_28_N_4_O_6_: 456.20, found: [M + H]^+^ 457.2. **HPLC:** 96.4% (254 nm).

### 3.5 Method Details

#### 3.5.1 Lifespan Assay

Age‐synchronized *C. elegans* were prepared in liquid medium (S‐complete medium with 50 mg/mL carbenicillin and 0.1 mg/mL fungizone) in flat‐bottom, optically clear 96‐well plates (Corning, Cat#351172) containing 150 μL total volume per well, as previously described (Clay and Petrascheck 2020; Kwon and Lee 2025). Plates contained ~10 animals per well in 6 mg/mL OP50. All experiments were performed with X‐ray‐irradiated OP50. Age‐synchronized animals were seeded as L1 larvae and grown at 20°C. Plates were covered with sealers to prevent evaporation. To prevent self‐fertilization, FUDR (0.12 mM final) was added 42–45 h after seeding. Drugs were added on the days indicated, and survival was scored manually by visualizing worm movement using an inverted microscope 3×/week. When used, DMSO was kept to a final concentration of 0.33% v/v. Statistical analysis was performed using the Mantel–Haenszel version of the log‐rank test as outlined in Petrascheck and Miller (2017) (Min and Lee 2025).

#### 3.5.2 O‐Propargyl Puromycin Translation

HEK293 (6 × 10^5^) or HT22 (5 × 10^3^) cells were seeded into black 96‐well plates (Costar, Cat#3603) with complete media (100 μL/well) and incubated overnight. For drug treatment, drugs were prepared at a 20 mM stock in 100% DMSO. This was further diluted with 12.5% DMSO/PBS to make 12× working solutions at the indicated concentrations. Four microlitre were added for a final concentration of 0.5% DMSO/PBS in each well. 0.5% DMSO/PBS served as a control. Drugs and control were added in triplicate and incubated for 2 h. Protein synthesis rates were determined using EZClick Global Protein Synthesis Assay Kit (BioVision, Cat#76305‐296) according to the manufacturer's protocol. In short, nascent polypeptides were pulse‐labeled by the addition of 1 μL 100× “protein label” and incubated for 30 min at 37°C. We included a 1 μL addition of DMSO as a “no‐protein label” control to subtract background fluorescence. Then the culture media was removed, and cells were washed once with 100 μL PBS. Cells were fixed by the addition of 100 μL “fixative solution” and incubated for 15 min on ice in the dark. This was aspirated off, and 100 μL “permeabilization buffer” was added and incubated for 10 min at room temperature. Buffer was removed and 20 μL fresh “permeabilization buffer” was added. The 1× EZclick reaction cocktail was prepared according to the protocol: 3 μL PBS, 1 μL copper reagent, 1 μL fluorescent azide, 5 μL reducing agent, scaled for the number of reactions needed. One hundred microlitre 1× EZclick reaction cocktail was added to each well and incubated. After 30 min, the reaction mixture was aspirated, and the cells were washed three times with 100 μL “wash buffer.” Wells were then incubated with 1× DAPI stain for 15 min and washed three times with “wash buffer.” As a positive control, 0.5 μM cycloheximide (CHX) was added 30 min prior to the addition of “protein label.” When thapsigargin was used, it was added at the same time as the tetracyclines at 250 nM.

Each well was imaged using the ImageExpress HT.ai confocal high‐content imaging system (IXM, Molecular Devices). A 20× Apo LWD 9na 0.95 water immersion objective was used with a confocal 50 μm slit setting. Laser power was set to 2%, and each image frame was exposed for 100 ms for GFP channel and 10 ms for the DAPI channel. Each well was imaged in four center locations. Images were analyzed using a custom Cell Profiler pipeline that autodetects an ROI based on DAPI staining of nuclei, then expands the ROI by 20 pixels, and then measures GFP signal. Measuring GFP/DAPI signal gives an approximation of total protein synthesis relative to the total number of cells seeded in each well.

#### 3.5.3 Oxytosis Ferroptosis Survival Assay—HT22 Cells

4 × 10^3^ cells were seeded into sterile black 96‐well plates with complete media and incubated overnight (100 μL/well). For drug treatment, drugs were prepared at a 10 mM stock in 100% DMSO. This was further diluted with 5% DMSO/PBS to make 25× working solutions. Four microlitre were added for a final concentration of 0.5% DMSO in each well. 0.2% DMSO served as a control. Drugs and control were added 2 h prior to the addition of oxytosis/ferroptosis inducers. Five millimolar glutamate (ThermoFisher, Cat#156212500) was added to each well, and cell viability was measured using CellTiter‐Glo (Promega, Cat#G9243). Luminescence was measured using BioTek Cytation 5 (Agilent). Results are shown as a percentage of untreated control cells. In experiments where ISRIB was used to block the ISR, 300 nM ISRIB was added 2 h prior to the addition of drugs/DMSO controls. When thapsigargin was used, it was added at the same time as the tetracyclines at 250 nM.

#### 3.5.4 Oxytosis Ferroptosis Survival Assay—Primary and iNeurons

##### 3.5.4.1 For Primary Neurons

Hippocampal primary neurons were prepared in sterile black 96‐well plates coated with borate buffer as described above. At DIV7, drugs were added at increasing concentrations to each well and incubated for 4 h. Then, 300 nM RSL3 (Selleck Chem, Cat#S8155) was added to each well and incubated for 24 h. Viability was determined using CellTiter‐Glo as above. When ISRIB was used, 300 nM was added to each well and incubated for 4 h prior to drug/control treatment.

##### 3.5.4.2 For iNeurons

On day 3, 24 h after puromycin selection, cells from a 6‐well plate were replated equally into 96‐well plates, at 2 × 10^4^, leaving the edge wells empty. Forty nanomolar BRDU was added to select against the remaining dividing iPSCs. The media was changed on days 7 and 10 with neuronal differentiation factors (BDNF, GDNF, NT3, Laminin). Cells were maintained with half‐media changes twice a week and were then allowed to grow until day 24. Then 0, 0.14, 0.41, 1.2, 3.7, 11, 33, or 100 μM 4‐epiminocycline was added to six wells of the 96‐well plate. After 2 h, 2 μM RSL3 was added to each well to induce ferroptosis. Twenty‐four hours later, viability was determined using CellTiter‐Glo as above.

#### 3.5.5 Surface Sensing of Translation (SUNSET)

##### 3.5.5.1 For *C. elegans*

Day 1 adult N2 worms were bleached, and eggs were allowed to hatch in S‐complete by shaking overnight. The next day, 10,000 L1 worms were seeded in a 10 cm plate containing a total volume of 20 mL S‐complete with 6 mg/mL OP50 bacteria, 50 μg/mL carbenicillin, and 0.1 μg/mL amphotericin B. Four millilitre FUDR (0.6 mM stock in S‐complete) was added to worms at the L4 stage in each plate. Two hours later, 100 μM of each compound was added, except 4‐epiminocycline and 12‐aminominocycline, which were added at 33 and 200 μM, respectively. After 12 h, worms were transferred into a 15 mL Corning tube containing a total volume of 4 mL S‐complete with 500 μL 6 mg/mL OP50 bacteria, 0.5 mg/mL puromycin (ThermoFisher, Cat#A11138‐03), and the same tetracycline concentrations used during the 12‐h pretreatment. After rotating the corning tubes for 4 h, worms were collected into 2 mL cryotubes by washing them with M9 once and with cold PBS three times. Worms were flash frozen in liquid nitrogen, and 150 μL of cold lysis buffer (20 mM Tris base, 100 mM NaCl, 1 mM MgCl_2_, pH 7.4, with protease inhibitors [Roche, Cat#11836153001]) was added, and samples were subsequently broken with a beak mill homogenizer (Fisher). Protein concentrations were determined by the Bradford protein assay (Bio‐Rad, Cat#5000006). Fifty microgram protein from each sample was loaded for western blot analysis using antibodies against puromycin (Millipore, Cat#MABE343) and GAPDH (Proteintech, Cat#10494‐1‐AP). Primary antibodies were diluted 1:5000 in 5% nonfat milk in TBST, and secondary antibodies were diluted 1:10,000. To determine the relative intensities of each blot, the integrated intensity was measured for each full anti‐puromycin lane using ImageJ. A similarly sized band with no signal was used to calculate and subtract background, and then each intensity was normalized to the corresponding GAPDH loading control. Finally, each condition was normalized to the DMSO control for quantification and statistics.

##### 3.5.5.2 For Primary Neurons

Cortical primary neurons were prepared in 10 cm dishes as described above. On DIV7, dishes were treated with 300 nM ISRIB or DMSO. Immediately after, tunicamycin was added to a final concentration of 3 μg/mL. Either 30 min or 18 h after tunicamycin addition, puromycin was added to a final concentration of 2 μg/mL. Two hours after puromycin addition, total protein was extracted with RIPA buffer (150 mM NaCl [Sigma‐Aldrich, Cat#S7653], 5 mM EDTA [Applichem, Cat#A1104], 50 mM Tris [ThermoFisher, Cat#BP152‐5], 1% NP‐40 [Millipore Sigma, Cat#492016], 0.5% sodium deoxycholate [ThermoFisher, Cat#BP349‐100], and 0.1% SDS) supplemented with protease and phosphatase inhibitor cocktail (ThermoFisher, Cat#78442). Protein concentrations were determined by the Bradford protein assay. Fifteen microgram from each sample was loaded for western blot analysis using antibodies against puromycin diluted 1:1000 in 5% nonfat milk in TBST. Relative intensities were determined as described above. Total protein was visualized with Coomassie stain solution (0.1% Coomassie Brilliant Blue R stain [ICN Biomedicals, Cat#02190343‐CF], 7% acetic acid, 50% methanol).

#### 3.5.6 Antibiotic Activity Assay

One colony of OP50 was inoculated overnight in a 37°C shaker in 5 mL LB media containing 50 μg/mL ampicillin. Fourteen to sixteen hours later, the pre‐inoculum was diluted 1:200 in 5 mL LB containing 50 μg/mL ampicillin. In a flat‐bottom, clear 96‐well plate, 100 μL of the diluted inoculum was added to each well being tested, one column (8 wells) per condition. DMSO or the indicated drugs were added, and a 0 time point was recorded for baseline OD_600_ values using a plate reader (Tecan Safire II). The plate was returned to the 37°C shaker, and OD_600_ was remeasured every hour. OD_600_ values for each time point were averaged across each well per condition.

#### 3.5.7 Zn^2+^ Chelation Assay

Chelation assay was performed using the Abchem Zinc Assay Kit (Abcam, Cat#ab102507). First, the included zinc standard was added to each well according to the manufacturer's protocol. In a clear 96‐well plate, samples were preincubated with 0, 100, 300, 780, 1200, and 1800 μM of each tetracycline in duplicate. Samples were allowed to incubate for 30 min before the determination of free Zn^2+^. Absorbance was measured at OD_560nm_ on the Tecan Safire II. EDTA was included as a positive control, and data were normalized with 0% remaining free Zn^2+^ defined by assay buffer with no zinc standard, and 100% free zinc being the DMSO control.

#### 3.5.8 MMP9 Inhibition

MMP9 inhibition was determined using a quenched fluorogenic peptide included in the MMP9 Inhibitor Screening Assay Kit following the manufacturer's protocol (Abcam, Cat#ab139449). N‐hydroxy‐2‐phenylethanamide (NNGH, 1 μM) was included as a positive control. In the screening assay, 100 μM of each tetracycline in triplicate was added to a well of a 96‐well plate containing MMP enzyme and assay buffer and allowed to incubate for 10 min. MMP9 substrate was then added to each well using a multichannel pipette, the plate was incubated for 60 min at 37°C, and fluorescence was measured using the BioTek Cytation 5. An identical procedure was followed for the generation of dose response curves for 12‐aminominocycline, 4‐epiminocycline, minocycline, and tigecycline, except concentrations were: 0, 1, 10, 30, 100, and 300 μM. Four technical replicates were used for each concentration, and the assay was repeated twice.

#### 3.5.9 UPR^MT^ Stress Imaging

20,000 SJ4100 [zcIs13 [*hsp‐6p::GFP*]], age‐synchronized animals were prepared as in the SUNSET method. At the developmental stage of L2, 100 μM of each tetracycline or DMSO was added and allowed to incubate for 12 h. After the treatment window, animals were washed 3× with S‐complete to remove any bacteria and suspended in 10 mL S‐complete. GFP intensity was quantified using the COPAS Biosorter (Union Biometric). Integral values for the GFP channel were used to determine fluorescent values. The animals were also sorted into 96‐well plates and imaged using an ImageXpress Micro XL High‐Content screening system (Molecular Devices) with a 2× objective.

#### 3.5.10 HSR Stress Imaging

20,000 CL2070 [*dvIs70* [*hsp‐16.2p::GFP + rol‐6(su1006)*]] animals were prepared as in the SUNSET method. One hundred micromolar of each tetracycline or DMSO was added to the liquid culture at the late L4 stage. On day 1 of adulthood, after 12 h of treatment, the liquid culture was transferred to a 50 mL Falcon tube, and worms were settled by gravity. The supernatant was aspirated off, leaving the concentrated worm pellet. The pellet was washed twice with S‐complete, pelleted by gravity again, and then transferred to a 10 cm NGM plate. Once the liquid was completely dry on the NGM plate, the plates were transferred to a 36°C incubator, upside down, and incubated for 1.5 h. Following heat shock, NGM plates were transferred to a 20°C incubator and allowed to recover for 8 h. Worm GFP intensity was determined as in the *hsp‐6::GFP* reporter using the COPAS Biosorter, but instead of being sorted into 96‐well plates, 5000 animals were bulk‐sorted into 2 mL cryotubes and flash frozen for RT‐qPCR analysis.

#### 3.5.11 Quantitative Real‐Time PCR (qRT‐PCR) and Data Analysis

All qRT‐PCR experiments were conducted according to the MIQE guidelines, except that samples were not tested in a bio‐analyzer, but photometrically quantified using a Nanodrop. Worm pellets were obtained from the HSR stress imaging experiment above. To extract RNA, frozen animals were suspended in ice‐cold Trizol (Qiagen, Cat#79306), then zirconium beads were added, and animals were broken open with a beak mill homogenizer (Fisherbrand). Following chloroform extraction, RNA was precipitated using isopropanol and washed once with 75% ethanol, followed by DNase (Sigma‐Aldrich, Cat#AMPD1‐1KT) treatment. Reverse transcription was carried out using iScript RT‐Supermix (Bio‐Rad, Cat#170–8841) at 42°C for 30 min. Quantitative PCR reactions were set up in 384‐well plates (Bio‐Rad, Cat#HSP3901), which included 2.5 μL Bio‐Rad SsoAdvanced SYBR Green Supermix (Cat#172–5264), 1 μL cDNA template (2.5 ng/μL, to final of 0.5 ng/μL in 5 μL PCR reaction), 1 μL water, and 0.5 μL of forward and reverse primers (150 nM final concentration). Quantitative PCR was carried out using a Bio‐Rad CFX384 Real‐Time thermocycler (95°C, 3 min; 40 cycles of 95°C 10 s, 60°C 30 s; Melting curve: 95°C 5 s, 60°C–95°C at 0.5°C increment, 10 s). Gene expression was normalized to three reference genes for *C. elegans* samples: *act‐1*, *xpg‐1*, and *rpl‐6*.

#### 3.5.12 Fluorescent Noncanonical Amino Acid Tagging (FUNCAT)

##### 3.5.12.1 For iNeurons

On day 3, 24 h after puromycin selection, cells from a 6‐well plate were replated equally into 6‐well plates, at 6 × 10^5^. On day 14, 0, 10, 30, or 100 μM 4‐epiminocycline or 12‐aminominocycline was added to each well of a 6‐well plate (3 mL media). Two hundred and fifty nanomolar CHX was added as a positive control in one of the wells. Drugs were prepared from 30 mM stock solutions in 100% DMSO. Working solutions were prepared at 300× in 15% DMSO and 20 μL was added to each well for a final concentration of 0.1% DMSO and allowed to incubate for 2 h. A 200 mM azidohomoalanine (AHA, Vector Laboratories, Cat#CCT‐1066‐1000) stock solution was prepared with Ultrapure H_2_O and brought to pH 7 with 10 N NaOH. Two hundred millimolar AHA was diluted in complete culture media pre‐warmed to 37°C for a final concentration of 8 mM. Media was replaced with AHA and pulse labeled for 2 h, after which labeling was stopped by removal of AHA media.

iNeurons were washed once in 1× DPBS and then lysed in a dish with the addition of 100 μL 10% RIPA/DPBS buffer + 1× protease inhibitors (Pierce Protease Inhibitor Mini Tablets, EDTA‐free, ThermoFisher, Cat#A32955) on ice. Cells were scraped into an Eppendorf tube and centrifuged at 5000 × *g* for 2 min to remove debris. Lysate was collected, and protein concentration was determined by Pierce BCA assay kit (ThermoFisher, Cat#23235) following the manufacturer's instructions.

Cell lysates were adjusted to 1.0 mg/mL using cold DPBS. To each sample (50 μL) in a PCR Eppendorf tube, 6 μL of freshly prepared click reaction mixture was added. The cocktail was prepared by the addition of 3 μL THPTA (1.7 mM in H_2_O), 1 μL CuSO_4_ (50 mM in H_2_O), 1 μL TAMARA‐azide (1.25 mM in DMSO), and 1 μL ascorbate (50 mM in H_2_O, prepared last). After the addition of the mixture, each reaction was vortexed, briefly centrifuged, and incubated at 40°C for 1 h. The reaction was quenched by the addition of 17 μL of 4× SDS loading buffer (Bio‐Rad, Cat#1610747), and heated at 95°C for 5 min. Thirty microgram protein was loaded per lane of a polyacrylamide gel (Bio‐Rad, Cat#4569034) and visualized by in‐gel fluorescence on a ChemiDoc MP flatbed fluorescence scanner (Bio‐Rad, Cat#12003154). After imaging, gels were stained with Coomassie blue to determine total protein. Gels were quantified in ImageJ by dividing the integrated intensity of each lane of the fluorescent image by the integrated intensity of the corresponding total protein lane after subtracting a similar area background lane, then normalizing to the DMSO control. Abbreviations: THPTA ((tris‐hydroxypropyltriazolylmethyl)amine; Combi‐blocks, Cat#QH‐3278); CuSO_4_, (copper(II) sulfate; Sigma‐Aldrich, Cat#C1297); Azide‐fluor 545 (5‐carboxytetramethylrhodamine‐azide; Millipore Sigma, Cat#760757); and ascorbate ((+) sodium L‐ascorbate; Sigma‐Aldrich, Cat#A7631).

##### 3.5.12.2 For In Vivo Translation

We utilized the in vivo translation measurement method developed in Xie et al. (Dong et al. 2026) but adapted for gel‐based FUNCAT. Ten‐week‐old male C57BL/6 mice were treated with either 4‐epiminocycline or control in drinking water (0.6 mg/mL) for 3 days (*n* = 6 per group). This dose was selected because it is well tolerated in mice, and we determined plasma levels of approximately 7 μM for the analog minocycline, which is comparable to circulating levels in human patients of 2–11 μM (Petrascheck and Miller 2017; Xie et al. 2025). On the 4th day, 100 mg/kg 4‐epiminocycline or control (saline) was administered via intraperitoneal (i.p.) injection. Thirty minutes later, AHA (dissolved in PBS) was i.p. injected at 50 mg/kg. Again, 30 min later, 50 mg/kg pentylenetetrazol (PTZ, dissolved in PBS) was i.p. injected. Ninety minutes later, the animals were sacrificed, PBS‐perfused, the hippocampus was harvested, and the samples were snap frozen in liquid nitrogen.

Samples were resuspended in 150 μL 1× DPBS and homogenized with a handheld tip sonicator (Fisher Scientific, sonic dismembrator model 100). Protein concentration of the homogenate was determined by the BCA protein assay kit. Protein lysate with AHA‐labeled nascent proteins was normalized by total protein to 2.0 mg/mL, and the click reaction with TAMRA‐azide was performed as described above.

#### 3.5.13 Rat Pharmacokinetic Determination

Pharmacokinetic testing for 4‐epiminocycline was conducted by Pharmaron. 4‐epiminocycline was formulated in PBS and was i.p. injected at a low dose of 25 mg/kg and a high dose of 50 mg/kg. A total of 3/3 male rats were assessed at 0.083, 0.25, 0.5, 1, 2, 4, and 8 h timepoints for blood. At the 8 h timepoint, the rats were sacrificed after blood collection, and the brains were weighed and then homogenized. Analytes from each collection were extracted and run on an LC–MS/MS (AB API 55000) to quantify compound concentration. Data were collected as plasma (ng/mL) and brain (ng/g) concentrations determined by an internal standard method.

#### 3.5.14 LC–MS Stability Analysis

Stability of 4‐epiminocycline and 12‐aminominocycline was assessed by LC–MS. Stock solutions of each compound were prepared at 30 mM in DMSO and either analyzed fresh or stored at room temperature for the indicated time points. At each time point, samples were diluted into HPLC water to a final concentration of 50 μM and analyzed by LC–MS using extracted ion chromatograms corresponding to the expected parent ion of each compound (457.2 for 12‐aminominocycline and 458.1 for 4‐epiminocycline). Parent compound stability was evaluated by monitoring the parent ion signal at the expected retention time over time. Extracted ion chromatogram intensities were scaled to the freshly prepared sample or day 0 sample for each compound. Visual changes in stock solution color were also documented after 7 days of room‐temperature storage. This analysis showed loss of the 12‐aminominocycline parent ion signal over time, whereas the 4‐epiminocycline parent ion signal remained comparatively stable across the measurement period.

## Author Contributions

K.J.C., S.S., and A.T. conducted the lifespan experiments (Figures 1A,B and 4C,D,F, Figure S2D,F,G). K.J.C. conducted the bacterial growth experiments (Figure 1C), the HT22 and HEK293 cell culture experiments (Figures 2 and 3), the LC–MS stability study (Figure S3A,B), and the biochemistry experiments (Figures 5D and 6D, Figures S1 and S3C,D). N.N. and A.P.V.E. conducted the iPSC and iNeuron experiments under the supervision of H.C. (Figure 5). I.N. conducted the primary neuron experiments (Figure 6F, Figure S3F). M.S.‐A. and A.T. conducted the mouse experiments (Figure 6), exposing mice to 4‐epiminocycline and harvesting their hippocampus. M.P. led the study, and M.P. and K.J.C. wrote the paper.

## Funding

This work was supported by NIH (R01AG079517, R21NS107951) and Helen Dorris Foundation.

## Conflicts of Interest

M.P. and K.J.C. are the scientific founders of Cyclone Therapeutics, a company developing nonantibiotic tetracyclines for therapeutic purposes. All other authors declare no conflicts of interest.

## Supporting information


**Figure S1:** MMP 9 inhibition is not required for tetracycline‐induced neuroprotection. (A) Shown is the general structure of the tetracyclines with the keto‐enol system (pink) at C11 and C12 that is responsible for the chelation of Zn^2+^ and other divalent ions. Structural modifications in 12‐aminominocycline and R464 disrupt the chelation center (blue and orange circles). (B) Colorimetric assay measuring the % remaining Zn^2+^ as a function of tetracycline concentration, indicative of the chelation effect of each tetracycline. 12‐aminominocycline does not chelate ions at concentrations up to 1800 μM, whereas 300 μM is required for R464. Other tetracyclines tested (gray) include: 4‐epiminocycline, minocycline, Col‐3, doxycycline, tigecycline, and evaracycline. EDTA was used as a positive control. (C) Bar graph shows the % remaining recombinant MMP9 activity after tetracycline treatment (100 μM). The assay measures proteolytic cleavage of a fluorogenic substrate, released upon cleavage. NNGH is a broad‐spectrum inhibitor of matrix metalloproteinases and was used as a positive control. Significance was determined by one‐way ANOVA with Dunnett's multiple comparisons, where ***p < 0.001 and ****p < 0.0001. Error bars indicate mean ± SD from three independent trials. (D) Dose response curve of four neuroprotective tetracyclines. Tetracyclines with IC_50_ > 300 μM are indicated as “not determined” (n.d.).
**Figure S2:** Tetracyclines elicit stress response‐dependent and independent longevity mechanisms. (A) Scatter plot shows the fold induction relative to DMSO of the *hsp16.2::GFP* heat shock response reporter after tetracycline treatment, followed by a 1.5 h, 35°C heat shock (HS). (B) qRT‐PCR quantification of relative GFP mRNA expression from untreated or tetracycline‐treated animals with and without a 1.5 h, 35°C HS. Tetracycline treatment suppresses the HS‐induced GFP fluorescence of the *hsp‐16.2p::GFP* reporter at the protein but not at the mRNA level. (C) qRT‐PCR quantification of endogenous *hsp16.2* mRNA expression following HS with or without preincubation of tetracycline treatment of wild‐type (N2) animals. (D) Survival plot of ISR‐deficient *eIF2*⍺*(rog3)* mutants, which lack the eIF2⍺ phosphorylation site. Only tetracyclines, but not salubrinal treatment, extend lifespan. (E) Quantification of three biological replicates from the SUNSET experiment in Figure 4E shows that 4‐epiminocycline and 12‐aminominocycline do not depend on *eIF2*⍺ phosphorylation for translation inhibition, while salubrinal does. (F) Survival plot of N2 and the partially ISR‐deficient *atf‐4(ok576)* mutant treated with DMSO or doxycycline. ATF‐4 is partially required for lifespan extension by doxycycline. The survival curve is one of the three trials quantified in G. (G) Comparison of the mean lifespan extension of N2 and *atf‐4(ok576)* animals treated with the indicated tetracycline. Doxycycline specifically loses efficacy in *atf‐4(ok576)* mutants across three biological replicates. Statistics for B, C, G: Significance was determined by one‐way ANOVA with Dunnett's multiple comparisons, where **p < 0.01, ***p < 0.001, ****p < 0.0001. All error bars indicate mean ± SD from three independent trials. Significance for all survival data (D, F) was determined by the log‐rank test.
**Figure S3:** Physicochemical properties of atypical tetracyclines. (A) Stock solutions of 12‐aminominocycline and 4‐epiminocycline that were either freshly prepared or stored at room temperature for 7 days. Photographs show a strong visual color change of 12‐aminominocycline, but not 4‐epiminocycline, consistent with reduced solution stability. (B) Extracted ion chromatograms (EICs) from LC–MS analysis of 4‐epiminocycline (red) and 12‐aminominocycline (blue) at the indicated time points. 12‐aminominocycline showed degradation and loss of the parent ion signal at the expected retention time, consistent with the degradation of 12‐aminominocycline. The EIC of 4‐epiminocycline EIC remained relatively unchanged, consistent with a long‐term stability in solution. The *y*‐axis shows intensity scaled to day 0, and the *x*‐axis shows retention time. (C) 4‐epiminocycline did not alter the ratio of nuclear–encoded (ATP5A) to mitochondrial‐encoded (MT‐CO1) electron transport chain proteins. (D) Quantification of ATP5A to MT‐CO1 ratio. Actin was used as a loading control for normalization between samples before comparison of nuclear/mitochondrial–encoded proteins. ns = *p* > 0.05. (E) Validation of ISRIB activity in Figure 6F. Primary neurons were co‐treated with 1 μM tunicamycin (Tm) to confirm the ability of ISRIB (300 nM) to rescue translation inhibited by tunicamycin. The 18 h timepoint shows significant inhibition of translation by Tm, which is rescued with ISRIB. (F) Survival of primary hippocampal neurons as a function of 12‐aminominocycline (blue) concentration after ferroptosis induction by RSL3 (300 nM). Co‐treatment with ISRIB (300 nM) does not abolish the neuroprotective effect, confirming ISR‐independence.

**Table S1:** Percent change in lifespan for each strain treated with the indicated tetracycline at the specified concentration (Cxn, μM). *p* values were calculated using the log‐rank test. The number of repeats (Rep.), defined as trials conducted on different dates or by different individuals, are indicated. The initial screen was not counted as a repeat and is left blank.

## Data Availability

The data that support the findings of this study are available in the Supporting Information of this article.
