# Supplementary material for: Atypical Tetracyclines Promote Longevity and Ferroptotic Neuroprotection via Translation Attenuation
Source: Aging Cell. 2026 Jun 18;25(6):e70587. doi: 10.1111/acel.70587 (PMC13278029; doi:10.1111/acel.70587)
Supplement: Supplementary file 1 — Figure S1: MMP 9 inhibition is not required for tetracycline‐induced neuroprotection. (A) Shown is the general structure of the tetracyclines with the keto‐enol system (pink) at C11 and C12 that is responsible for the chelation of Zn2+ and other divalent ions. Structural modifications in 12‐aminominocycline and R464 disrupt the chelation center (blue and orange circles). (B) Colorimetric assay measuring the % remaining Zn2+ as a function of tetracycline concentration, indicative of the chelation effect of each tetracycline. 12‐aminominocycline does not chelate ions at concentrations up to 1800 μM, whereas 300 μM is required for R464. Other tetracyclines tested (gray) include: 4‐epiminocycline, minocycline, Col‐3, doxycycline, tigecycline, and evaracycline. EDTA was used as a positive control. (C) Bar graph shows the % remaining recombinant MMP9 activity after tetracycline treatment (100 μM). The assay measures proteolytic cleavage of a fluorogenic substrate, released upon cleavage. NNGH is a broad‐spectrum inhibitor of matrix metalloproteinases and was used as a positive control. Significance was determined by one‐way ANOVA with Dunnett's multiple comparisons, where ***p < 0.001 and ****p < 0.0001. Error bars indicate mean ± SD from three independent trials. (D) Dose response curve of four neuroprotective tetracyclines. Tetracyclines with IC50 > 300 μM are indicated as “not determined” (n.d.). Figure S2: Tetracyclines elicit stress response‐dependent and independent longevity mechanisms. (A) Scatter plot shows the fold induction relative to DMSO of the hsp16.2::GFP heat shock response reporter after tetracycline treatment, followed by a 1.5 h, 35°C heat shock (HS). (B) qRT‐PCR quantification of relative GFP mRNA expression from untreated or tetracycline‐treated animals with and without a 1.5 h, 35°C HS. Tetracycline treatment suppresses the HS‐induced GFP fluorescence of the hsp‐16.2p::GFP reporter at the protein but not at the mRNA level. (C) qRT‐PCR quantification [file ACEL-25-e70587-s002.docx]

**
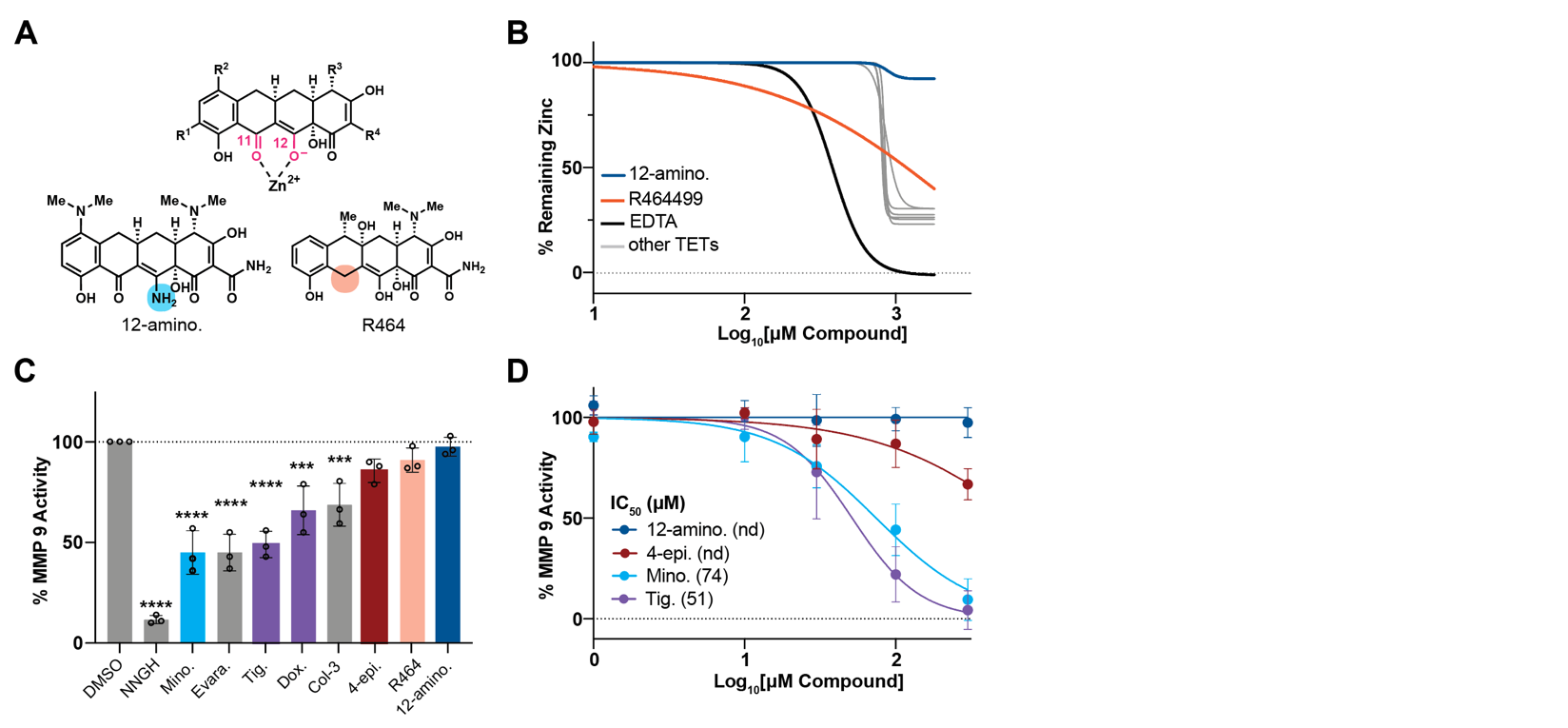
**

**Figure S1: MMP 9 inhibition is not required for tetracycline-induced neuroprotection**

**(A)** Shown is the general structure of the tetracyclines with the keto-enol system (pink) at C11 & C12 that is responsible for the chelation of Zn^2+^ and other divalent ions. Structural modifications in 12-aminominocycline and R464 disrupt the chelation center (blue & orange circles). **(B)** Colorimetric assay measuring the % remaining Zn^2+^ as a function of tetracycline concentration, indicative of the chelation effect of each tetracycline. 12-aminominocycline does not chelate ions at concentrations up to 1800 µM, whereas 300 µM is required for R464. Other tetracyclines tested (grey) include: 4-epiminocycline, minocycline, Col-3, Doxycycline, Tigecycline, & Evaracycline. EDTA was used as a positive control. **(C)** Bar graph shows the % remaining recombinant MMP9 activity after tetracycline treatment (100 µM). The assay measures proteolytic cleavage of a fluorogenic substrate, released upon cleavage. NNGH is a broad-spectrum inhibitor of matrix metalloproteinases and was used as a positive control. Significance was determined by one-way ANOVA with Dunnett's multiple comparisons, where *** = *p < 0.001* and **** = *p < 0.0001*. Error bars indicate mean ± SD from three independent trials. **(D)** Dose response curve of four neuroprotective tetracyclines. Tetracyclines with IC_50_ greater than 300 µM are indicated as "not determined" (n.d.).

**
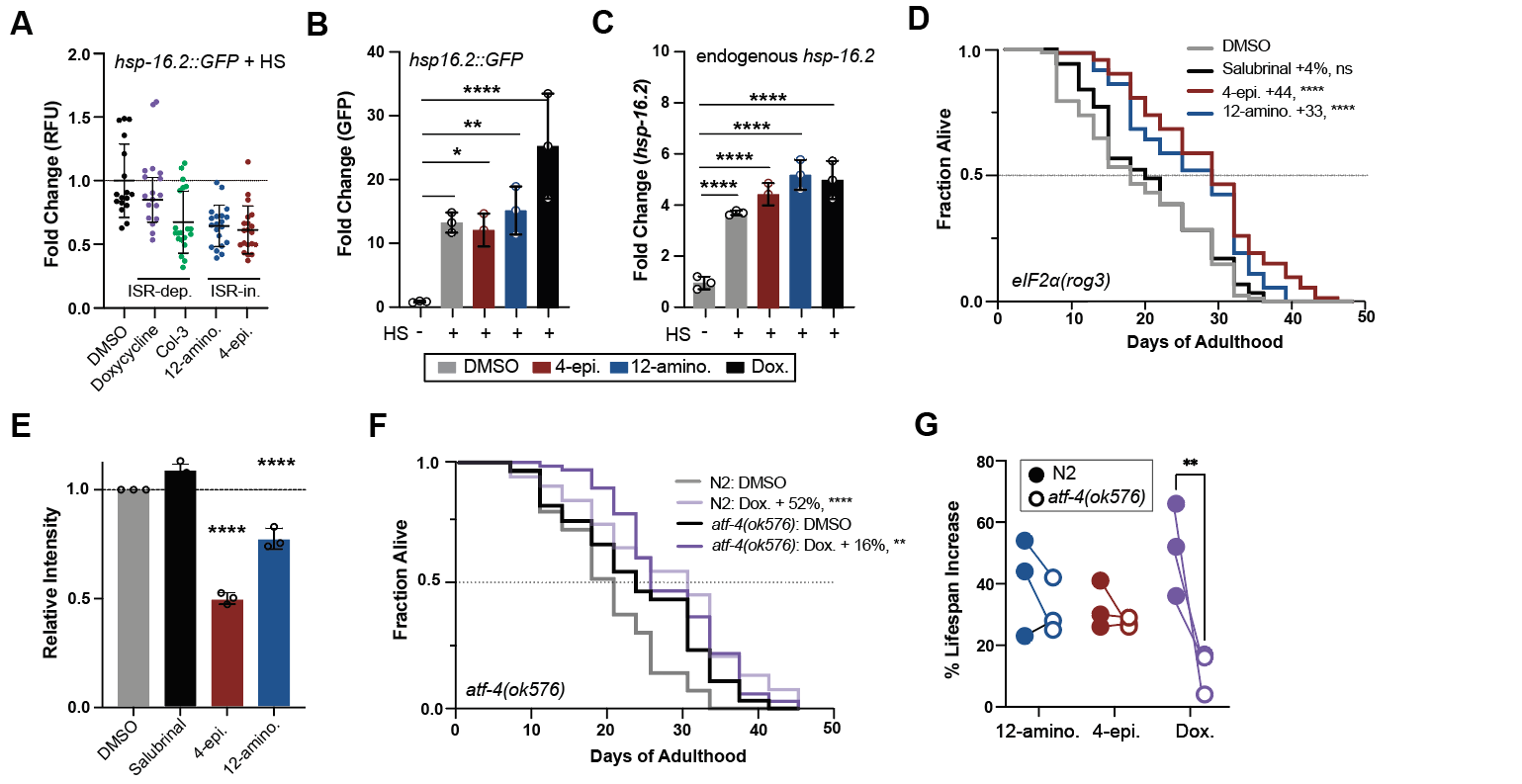
**

**Figure S2: Tetracyclines elicit stress response-dependent and independent longevity mechanisms**

**(A)** Scatter plot shows the fold induction relative to DMSO of the *hsp16.2::GFP* heat shock response reporter after tetracycline treatment, followed by a 1.5 hr, 35°C heat shock (HS)**. (B)** qRT-PCR quantification of relative GFP mRNA expression from untreated or tetracycline-treated animals with and without a 1.5 hr, 35°C HS. Tetracycline treatment suppresses the HS-induced GFP fluorescence of the *hsp-16.2p::GFP* reporter at the protein but not at the mRNA level. **(C)** qRT-PCR quantification of endogenous *hsp16.2* mRNA expression following HS with or without pre-incubation of tetracycline treatment of wild-type (N2) animals. **(D)** Survival plot of ISR-deficient *eIF2*⍺*(rog3)* mutants, which lack the eIF2⍺ phosphorylation site. Only tetracyclines, but not salubrinal treatment, extend lifespan. **(E)** Quantification of 3 biological replicates from the SUNSET experiment in Figure 4E shows that 4-epiminocycline and 12-aminominocycline do not depend on *eIF2*⍺ phosphorylation for translation inhibition, while salubrinal does. **(F)** Survival plot of N2 and the partially ISR-deficient *atf-4(ok576)* mutant treated with DMSO or doxycycline. ATF-4 is partially required for lifespan extension by doxycycline. The survival curve is one of the three trials quantified in Figure S2G. **(G)** Comparison of the mean lifespan extension of N2 and *atf-4(ok576)* animals treated with the indicated tetracycline. Doxycycline specifically loses efficacy in *atf-4(ok576)* mutants across 3 biological replicates. Statistics for **B**, **C**, **G**: Significance was determined by one-way ANOVA with Dunnett's multiple comparisons, where ** = *p < 0.01*, *** = *p < 0.001*, **** = *p < 0.0001*. All error bars indicate mean ± SD from three independent trials. Significance for all survival data (**D**, **F**) was determined by the log-rank test.

**
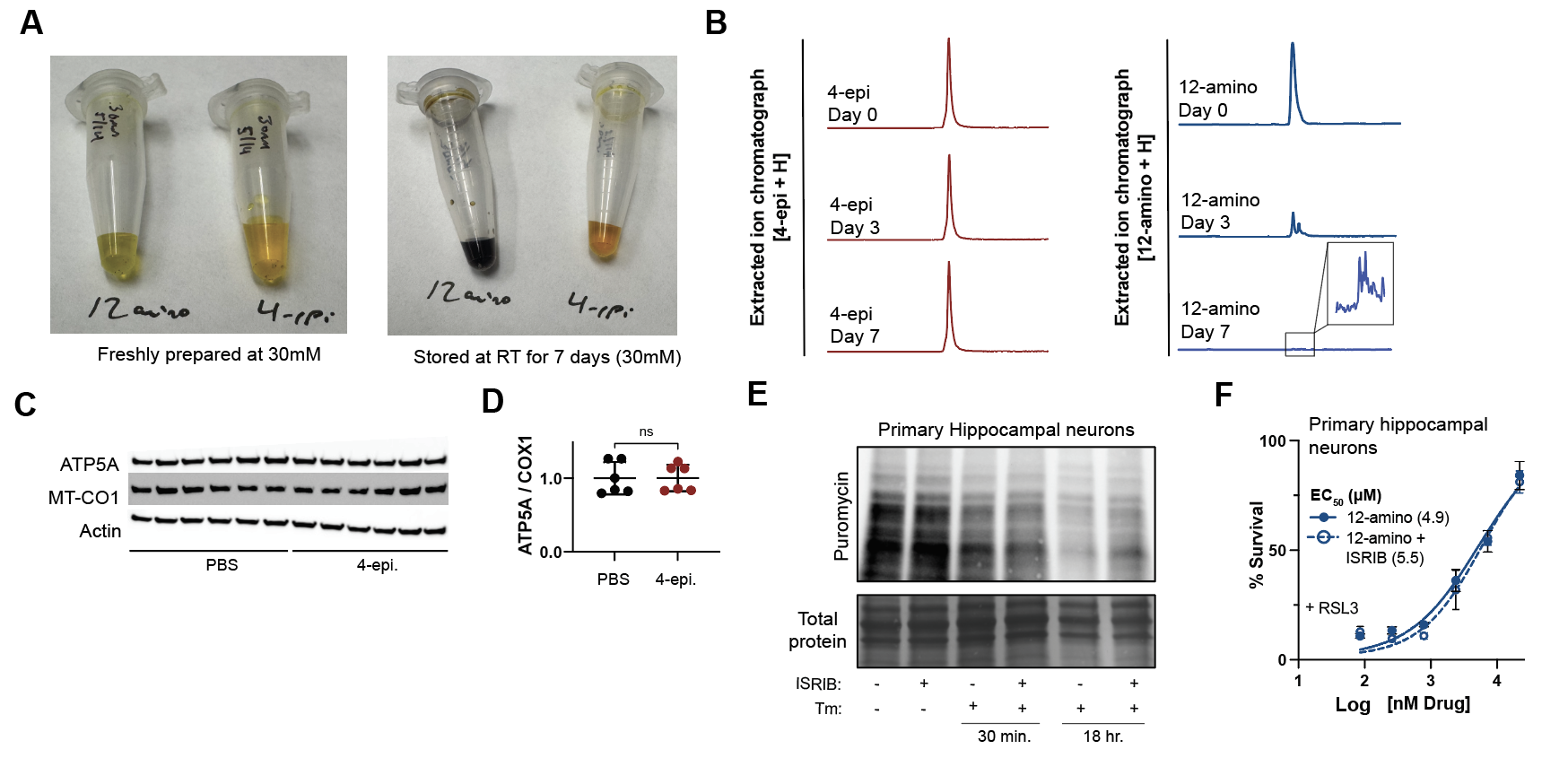
**

**Figure S3: Physicochemical properties of atypical tetracyclines**

**(A)** Stock solutions of 12-aminominocycline and 4-epiminocycline that were either freshly prepared or stored at room temperature for 7 days. Photographs show a strong visual color change of 12-aminominocycline, but not 4-epiminocycline, consistent with reduced solution stability. **(B)** Extracted ion chromatograms (EICs) from LC-MS analysis of 4-epiminocycline (red) and 12-aminominocycline (blue) at the indicated time points. 12-aminominocycline showed degradation and loss of the parent ion signal at the expected retention time, consistent with the degradation of 12-aminominocycline. The EIC of 4-epiminocycline EIC remained relatively unchanged, consistent with a long-term stability in solution. The y-axis shows intensity scaled to day 0, and the x-axis shows retention time. **(C)** 4-epiminocycline did not alter the ratio of nuclear–encoded (ATP5A) to mitochondrial-encoded (MT-CO1) electron transport chain proteins. **(D)** Quantification of ATP5A to MT-CO1 ratio. Actin was used as a loading control for normalization between samples before comparison of nuclear/mitochondrial–encoded proteins. ns = *p > 0.05*. **(E)** Validation of ISRIB activity in Figure 6F. Primary neurons were co-treated with 1 µM tunicamycin (Tm) to confirm the ability of ISRIB (300 nM) to rescue translation inhibited by tunicamycin. The 18 hr timepoint shows significant inhibition of translation by Tm, which is rescued with ISRIB. **(F)** Survival of primary hippocampal neurons as a function of 12-aminominocycline (blue) concentration after ferroptosis induction by RSL3 (300 nM). Co-treatment with ISRIB (300 nM) does not abolish the neuroprotective effect, confirming ISR-independence.
